# Development and Function of Invariant Natural Killer T Cells Producing T_H_2- and T_H_17-Cytokines

**DOI:** 10.1371/journal.pbio.1001255

**Published:** 2012-02-07

**Authors:** Hiroshi Watarai, Etsuko Sekine-Kondo, Tomokuni Shigeura, Yasutaka Motomura, Takuwa Yasuda, Rumi Satoh, Hisahiro Yoshida, Masato Kubo, Hiroshi Kawamoto, Haruhiko Koseki, Masaru Taniguchi

**Affiliations:** 1 Laboratory for Immune Regulation, RIKEN Research Center for Allergy and Immunology, Kanagawa, Japan; 2 PRESTO, Japan Science and Technology Agency, Tokyo, Japan; 3 Division of Biotechnology, Research Institute for Biological Science, Tokyo University of Science, Chiba, Japan; 4 Laboratory for Immunogenetics, RIKEN Research Center for Allergy and Immunology, Kanagawa, Japan; 5 Laboratory for Lymphocyte Development, RIKEN Research Center for Allergy and Immunology, Kanagawa, Japan; 6 Laboratory for Developmental Genetics, RIKEN Research Center for Allergy and Immunology, Kanagawa, Japan; National Jewish Medical and Research Center/Howard Hughes Medical Institute, United States of America

## Abstract

There is heterogeneity in invariant natural killer T (*i*NKT) cells based on the expression of CD4 and the IL-17 receptor B (IL-17RB), a receptor for IL-25 which is a key factor in T_H_2 immunity. However, the development pathway and precise function of these *i*NKT cell subtypes remain unknown. IL-17RB^+^ *i*NKT cells are present in the thymic CD44^+/−^ NK1.1^−^ population and develop normally even in the absence of IL-15, which is required for maturation and homeostasis of IL-17RB^−^ *i*NKT cells producing IFN-γ. These results suggest that *i*NKT cells contain at least two subtypes, IL-17RB^+^ and IL-17RB^−^ subsets. The IL-17RB^+^ *i*NKT subtypes can be further divided into two subtypes on the basis of CD4 expression both in the thymus and in the periphery. CD4^+^ IL-17RB^+^ *i*NKT cells produce T_H_2 (IL-13), T_H_9 (IL-9 and IL-10), and T_H_17 (IL-17A and IL-22) cytokines in response to IL-25 in an E4BP4-dependent fashion, whereas CD4^−^ IL-17RB^+^ *i*NKT cells are a retinoic acid receptor-related orphan receptor (ROR)γt^+^ subset producing T_H_17 cytokines upon stimulation with IL-23 in an E4BP4-independent fashion. These IL-17RB^+^ *i*NKT cell subtypes are abundantly present in the lung in the steady state and mediate the pathogenesis in virus-induced airway hyperreactivity (AHR). In this study we demonstrated that the IL-17RB^+^ *i*NKT cell subsets develop distinct from classical *i*NKT cell developmental stages in the thymus and play important roles in the pathogenesis of airway diseases.

## Abstract 2

Four distinct subsets of invariant natural killer T (NKT) cells are shown to differentiate in the thymus, then migrate to peripheral tissues where they retain their phenotypic and functional characteristics.

## Author Summary

T cells are a diverse group of immune cells involved in cell-mediated acquired immunity. One subset of T cells is the innate-like invariant natural killer T (*i*NKT) cells that recognize glycolipid ligands on target cells instead of peptides. We know that functionally distinct subtypes of *i*NKT cells are involved in specific pathologies, yet their development, phenotypes, and functions are not well understood. Here, we determine the relationship between various mouse *i*NKT cell subsets, identify reliable molecular markers for these subsets, and show that these contribute to their functional differences. We identify four *i*NKT cell subsets that we show arise via different developmental pathways and exhibit different cytokine profiles. Importantly, we show that these subsets can be isolated from the thymus (the organ of all T cells), as well as from peripheral tissues such as spleen, liver, lung, and lymph nodes. Contrary to the general understanding that *i*NKT cells mature after their exit from the thymus and their migration into peripheral tissues, we conclude that distinct phenotypic and functional *i*NKT cell subsets can be distinguished in the thymus by virtue of the presence or absence of the cytokine receptor IL-17RB and another cell surface molecule called CD4, and these subsets then migrate to peripheral tissues where they retain their phenotypic and functional characteristics. Regarding functional significance, we show that those *i*NKT cell subsets that lead to airway hyper-responsiveness to respiratory viruses are different to those that lead to allergen-induced airway hyperreactivity, which will enable researchers to focus on specific subsets as potential targets for therapeutic intervention.

## Introduction

Natural killer T (NKT) cells, unlike conventional T cells bearing diverse antigen receptors, are characterized by the expression of an invariant T cell receptor (TCR), Vα14Jα18 paired with Vβ8, Vβ7, or Vβ2 in mice [1] and the Vα24Jα18/Vβ11 pair in humans [2],[3], that recognizes glycolipid antigens in conjunction with the monomorphic MHC class I-like CD1d molecule [4],[5]. Therefore, these cells are termed invariant NKT (*i*NKT) cells. Another characteristic feature of *i*NKT cells is their rapid and massive production of a range of cytokines, such as those typically produced by T helper cell (T_H_) 1, T_H_2, and T_H_17 cells [6]–[8], upon stimulation with their ligand, α-Galactosylceramide (α-GalCer) [9],.

It is speculated that the ability of *i*NKT cells to produce these various cytokines is due either to the microenvironment in which they undergo priming or to the existence of functionally distinct subtypes of *i*NKT cells producing different cytokines; however, there is no clear-cut evidence to support the latter notion. It has been reported that *i*NKT cells include both CD4^+^ and CD4^−^ subtypes [6],[11], each of which produces different cytokines. Human CD4^+^ *i*NKT cells produce both T_H_1 and T_H_2 cytokines, whereas CD4^−^ *i*NKT cells produce mainly T_H_1 cytokines [12],[13]. Although such functional differences were originally less apparent in mouse CD4^+^ and CD4^−^ *i*NKT cells, two functionally distinct subtypes of *i*NKT cells in the mouse thymus have since been identified based on NK1.1 expression; NK1.1^−^ *i*NKT cells produce a large amount of IL-4 and little IFN-γ, whereas NK1.1^+^ *i*NKT cells produce less IL-4 and more IFN-γ [14],[15]. Furthermore, CD4^−^ *i*NKT cells in the liver have been found to be more effective in mediating tumor rejection than CD4^+^ *i*NKT cells in the liver or any other tissues [16].

There is also further heterogeneity of CD4^+^ *i*NKT cells in terms of expression of the IL-17 receptor B (IL-17RB), a receptor for IL-25 [17]. IL-25 is a key factor in T_H_2 immunity, including allergic reactions and airway hyperreactivity (AHR). The CD4^+^ IL-17RB^+^ *i*NKT cells produce large amounts of IL-13 and IL-4 but little IFN-γ in response to IL-25, mediating a key role in IL-25-driven AHR [17],[18]. Another subset of newly identified *i*NKT cells within the NK1.1^−^ CD4^−^ subset is the retinoic acid receptor-related orphan receptor (ROR)γt^+^ *i*NKT cells. These cells can induce autoimmune disorders by their production of IL-17A and IL-22 [8],[19], even though IL-17A-producing *i*NKT cells are not restricted to a particular *i*NKT cell subset [20].

The emergence of functionally distinct subpopulations of *i*NKT cells is reminiscent of how *i*NKT subtypes develop in the thymus and expand in the periphery. Here, we demonstrate IL-17RB^+^ *i*NKT cells are a subtype distinct from the CD122^+^ *i*NKT cells producing IFN-γ. Moreover, there are two subtypes of IL-17RB^+^ *i*NKT cells; CD4^−^ produces T_H_17 cytokines in an IL-23-dependent fashion, whereas the other CD4^+^ produces T_H_2 and T_H_17 cytokines in an IL-25 dependent manner. In addition, these IL-17RB^+^ *i*NKT cells contribute to the induction of virus and viral antigen-induced chronic AHR.

## Results

### Identification of Two Distinct Subtypes in *i*NKT Cells; IL-17RB^+^ *i*NKT Cells Producing T_H_2 and T_H_17 Cytokines and IL-17RB^−^ *i*NKT Cells Producing IFN-γ

**Figure 1:**
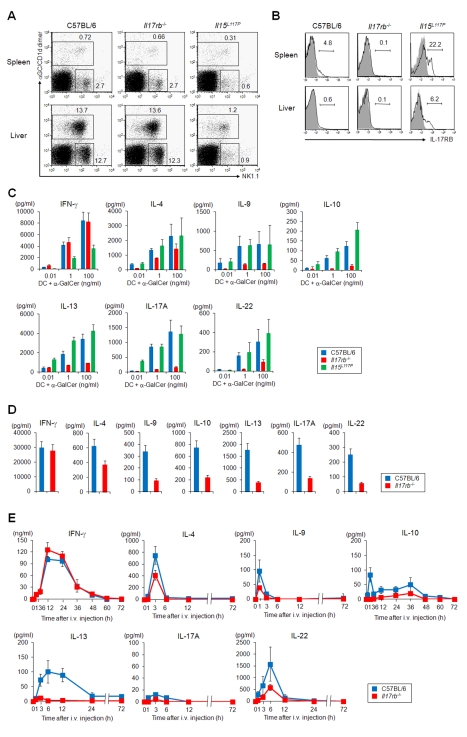
Function of *i*NKT cells in the spleen and liver from *Il17rb* ^−/−^ and *Il15* ^L117P^ mice. (A) FACS profile of spleen and liver mononuclear cells in WT, *Il17rb* ^−/−^ and *Il15* ^L117P^ mice on a B6 background. Numbers are percentage of gated cells. α-GalCer/CD1d dimer^+^ *i*NKT cells and α-GalCer/CD1d dimer^−^ NK1.1^+^ NK cells were slightly decreased in *Il17rb* ^−/−^ mice and markedly reduced in *Il15* ^L117P^ mice. (B) IL-17RB expression in spleen and liver *i*NKT cells of WT B6, *Il17rb* ^−/−^ and *Il15* ^L117P^ mice. Shaded profiles in the histograms indicate the background staining with isotype matched control mAb. (C, D) In vitro cytokine production by spleen *i*NKT cells from *Il17rb* ^−/−^ and *Il15* ^L117P^ mice (C) and by liver *i*NKT cells from *Il17rb* ^−/−^ mice (D). Sorted *i*NKT cells (5×10^4^/100 µL) from spleen and liver of WT B6 and *Il17rb* ^−/−^ mice were co-cultured with BM-DCs (5×10^3^/100 µL) for 48 h in the presence of the indicated doses of α-GalCer. The *Il17rb* ^−/−^ *i*NKT cells produced IFN-γ at levels equivalent to WT, while T_H_2 and T_H_17 cytokine production, except for IL-4, were severely impaired. (E) *i*NKT cell-dependent cytokine production in WT B6 and *Il17rb* ^−/−^ mice in vivo. α-GalCer (2 µg) was i.v. injected and the levels of cytokines in serum were analyzed at the indicated time points. The serum IFN-γ levels were similar in both mice, whereas production of T_H_2 and T_H_17 cytokines, except for IL-4, was significantly reduced in the *Il17rb* ^−/−^ mice. Cytokines were measured by ELISA or a cytometric bead array system at the indicated time points. Data are mean ± SDs from three mice and repeated three times with similar results.

We previously identified a fraction of splenic CD4^+^ *i*NKT cells that expresses IL-17RB and produces T_H_2 cytokines after treatment with IL-25 [17]. In order to directly analyze the function of IL-17RB on *i*NKT cells, we generated IL-17RB-deficient mice by the disruption of exon 1 and exon 2 of the *Il17rb* gene (Figure S1). We then compared the number and function of *i*NKT cells in the spleen and the liver from *Il17rb* ^−/−^ mice on a C57BL/6 (B6) background to those of wild type (WT) B6 mice. We also included in our comparison *Il15* ^L117P^ mice, in which leucine (CTT) at amino acid position 117 of IL-15 was substituted with proline (CCT), because IL-15 is reported to be critical for the development and homeostatic maintenance of *i*NKT cells [21],[22], as well as other cell types such as NK and CD8^+^ memory T cells [23],[24]. As shown in Figure 1A, the number of *i*NKT cells in *Il17rb* ^−/−^ mice was only slightly decreased in the spleen, and was almost comparable in the liver, compared with B6 mice, findings consistent with our previous analysis on the distribution of IL-17RB^+^ *i*NKT cells (3%–5% in the spleen and almost none in the liver) as detected by a specific monoclonal antibody [17]. Similarly, *Il15* ^L117P^ mice appear to recapitulate the previously reported phenotype of *Il15* ^−/−^ mice [21],[22] because *i*NKT and NK cells were decreased by 50% in the spleen and by 90% in the liver, indicating that the L117P mutation resulted in the loss of IL-15 function.

We then analyzed the frequency of IL-17RB^+^ subtypes among α-GalCer/CD1d dimer^+^ *i*NKT cells in the spleen and liver of WT, *Il17rb* ^−/−^, and *Il15* ^L117P^ mice (Figure 1B). The percentage of IL-17RB^+^ *i*NKT cells was increased more than 4 times in the spleen and 10 times in liver of the *Il15* ^L117P^ mice.

By using *Il17rb* ^−/−^ and *Il15* ^L117P^ mice, we further analyzed the *i*NKT cell subtypes in terms of their ability to produce cytokines (Figure 1C and 1D). α-GalCer/CD1d dimer^+^ TCRβ^+^ *i*NKT cells from the spleen of WT, *Il17rb* ^−/−^, and *Il15* ^L117P^ mice (Figure 1C) and those from the liver of B6 and *Il17rb* ^−/−^ mice (Figure 1D) were sorted and co-cultured with GM-CSF-induced bone marrow derived dendritic cells (BM-DCs) in the presence of α-GalCer. The *Il17rb* ^−/−^ *i*NKT cells produced normal levels of IFN-γ, but this was significantly decreased in *Il15* ^L117P^ *i*NKT cells. Intriguingly, there was impaired production of not only T_H_2 cytokines such as IL-9, IL-10, and IL-13, but also of T_H_17 cytokines IL-17A and IL-22 in *Il17rb* ^−/−^ *i*NKT cells, but not in *Il15* ^L117P^ *i*NKT cells in the spleen (Figure 1C and 1D), even though the number of *i*NKT cells were only slightly decreased in *Il17rb* ^−/−^ (see Figure 1A). The *i*NKT cells derived from WT, *Il17rb* ^−/−^, or *Il15* ^L117P^ failed to produce any indicated cytokines when co-cultured with BM-DCs from *Cd1d1* ^−/−^ mice (unpublished data), indicating the cytokine production from *i*NKT cells are absolutely CD1d/α-GalCer dependent.

To examine the functional activity of *Il17rb* ^−/−^ *i*NKT cells in vivo, we administered α-GalCer (2 µg) intravenously (i.v.) and monitored serum cytokine levels (Figure 1E). The production of IFN-γ peaked normally at 12 to 24 h after stimulation in the *Il17rb* ^−/−^ mice. On the other hand, the production (around 1–6 h) of other cytokines, such as IL-9, IL-10, IL-13, IL-17A, and IL-22, was severely impaired in the *Il17rb* ^−/−^ mice. The results suggest that IL-17RB^+^ *i*NKT cells are distinct from IL-17RB^−^ *i*NKT cells, which mainly produce IFN-γ, and also that IL-17RB^+^ *i*NKT cells produce IL-9, IL-10, and IL-13 among T_H_2 cytokines and IL-17A and IL-22 T_H_17-type cytokines.

### Development of IL-17RB^+^ and IL-17RB^−^ *i*NKT Subtypes in the Thymus

**Figure 2:**
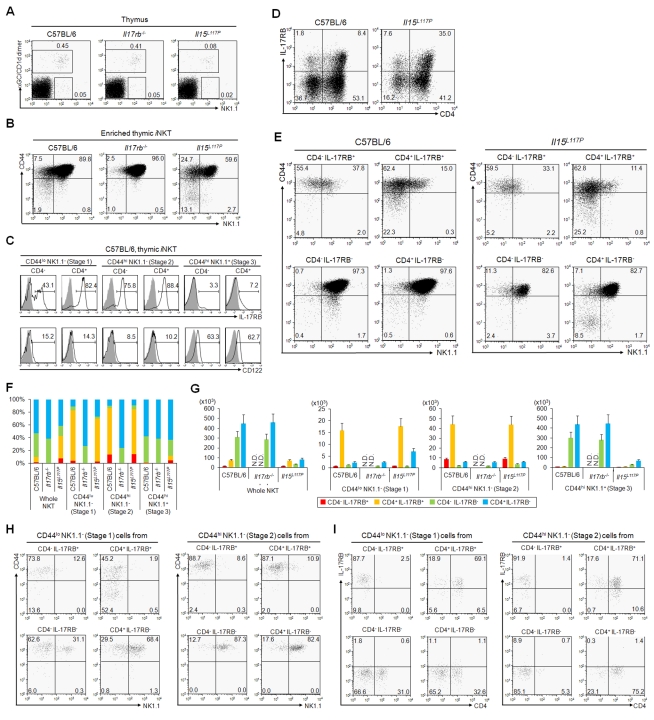
Profile of *i*NKT cells in the thymus of *Il17rb* ^−/−^ and *Il15* ^L117P^ mice. (A, B) FACS profiles of thymus (A) and enriched thymic *i*NKT cells (B) in WT, *Il17rb* ^−/−^ and *Il15* ^L117P^ mice on a B6 background. (A) α-GalCer/CD1d dimer^+^ *i*NKT cells were slightly decreased in *Il17rb* ^−/−^ mice and markedly reduced in *Il15* ^L117P^ mice. (B) There was a loss of the NK1.1^−^ population in *Il17rb* ^−/−^ thymic *i*NKT cells, while *Il15* ^L117P^ thymic *i*NKT cells showed impairment of the NK1.1^+^ population. (C) IL-17RB and CD122 expression by thymic *i*NKT cell populations of B6 mice. IL-17RB and CD122 expression in CD4^−^ and CD4^+^ of CD44^lo^ NK1.1^−^ (Stage 1), CD44^lo^ NK1.1^+^ (Stage 2), and CD44^hi^ NK1.1^+^ (Stage 3) populations were analyzed. IL-17RB expression was observed in Stages 1/2, while CD122 expression was in the Stage 3 cells. (D, E) Profiles of thymic *i*NKT cells in B6 and *Il15* ^L117P^ mice showing expression of IL-17RB and CD4 (D) and further divided into CD44 and NK1.1 subpopulations (E). The percentage of IL-17RB^+^ *i*NKT cells was increased due to the loss of expansion of IL17RB^−^ *i*NKT cells in *Il15* ^L117P^ mice. CD4^−^ and CD4^+^, IL-17RB^+^ *i*NKT cells were almost all Stage 1 and Stage 2 in both WT B6 and *Il15* ^L117P^ mice. On the other hand, the majority of CD4^−^ and CD4^+^, IL-17RB^−^ *i*NKT cells were Stage 3 in both WT B6 and *Il15* ^L117P^ mice. Loss of expansion of CD4^−^ and CD4^+^, IL-17RB^−^ *i*NKT cells was also observed in *Il15* ^L117P^ mice. (F, G) Percentage (F) and cell number (G) of the total *i*NKT cells and the four subtypes (i.e. IL-17RB^+/−^ and CD4^+/−^) in B6, *Il17rb* ^−/−^ and *Il15* ^L117P^ mice based on their CD44 and NK1.1 expression patterns. The number of CD4^−^ and CD4^+^, IL-17RB^−^ *i*NKT cells was significantly decreased especially in Stage 3 in *Il15* ^L117P^ mice compared to WT B6 mice. By contrast, CD4^−^ and CD4^+^, IL-17RB^+^ *i*NKT cells in *Il15* ^L117P^ mice were present in numbers comparable to WT. Results are representative of those from three independent experiments. (H, I) Development of *i*NKT subtypes in Stages 1 and 2. Stage 1 and 2 cells in the four *i*NKT subtypes (i.e. IL-17RB^+/−^ and CD4^+/−^) from WT B6 mice were sorted and cocultured with dGuo treated 15 dpc FT lobes from *Jα18* ^−/−^ mice (1,000 cells/well). 10 d after culture, cells were recovered and analyzed the surface expression pattern in CD44 versus NK1.1 (H) and CD4 versus IL-17RB (I). IL-17RB^−^ precursors gave rise through Stage 2 to Stage 3 cells with IL-17RB^−^, while IL-17RB^+^ subtypes gave rise to Stage 2 cells with IL-17RB^+^. Results are representative of those from three independent experiments.

*i*NKT cells in the spleen and liver from *il17rb* ^−/−^ mice are defective in the production of IL-9, IL-10, IL-13, IL-17A, and IL-22, while IFN-γ production is diminished in *Il15* ^L117P^ *i*NKT cells (Figure 1). We therefore attempted to identify the origin of IL-17RB^+^ *i*NKT cells in the thymus by comparing α-GalCer/CD1d dimer^+^ *i*NKT cells in B6 with those in *Il17rb* ^−/−^ and in *Il15* ^L117P^ mice on a B6 background (Figure 2A). The percentage and number of *i*NKT cells in the thymus were severely decreased in *Il15* ^L117P^ mice to a similar extent as previously reported in *Il15* ^−/−^ mice [21]. By contrast, the percentage and number of *i*NKT cells in *Il17rb* ^−/−^ mice was only slightly decreased, to a similar extent to that seen in the spleen and liver (Figure 1C and 1D). In order to analyze their phenotype precisely, enriched α-GalCer/CD1d dimer^+^ *i*NKT cells were further divided based on the expression of CD44 and NK1.1 (Figure 2B), because *i*NKT cells can be classified into developmental stages based on the cell surface expression of these molecules, i.e., CD44^lo^ NK1.1^−^ (Stage 1), CD44^hi^ NK1.1^−^ (Stage 2), and CD44^hi^ NK1.1^+^ (Stage 3) [14],[25]. In agreement with earlier results [21], there was a decrease in the CD44^hi^ NK1.1^+^ (Stage 3) population of α-GalCer/CD1d dimer^+^ *i*NKT cells in the thymus of *Il15* ^L117P^ mice. By contrast, the percentage and number of *i*NKT cells in *Il17rb* ^−/−^ mice were reduced, especially in the CD44^lo^ NK1.1^−^ (Stage 1) and CD44^hi^ NK1.1^−^ (Stage 2) populations, although the CD44^hi^ NK1.1^+^ (Stage 3) population was unchanged (Figure 2B). To determine whether the reduction in absolute numbers of developmental Stages 1 and 2 *i*NKT cell populations in *Il17rb* ^−/−^ mice is due to a developmental defect or to bypassing of these developmental stages, we analyzed surface expression of IL-17RB and CD122, a receptor for IL-15 (Figure 2C). Consistent with the observation shown in Figure 2B, IL-17RB expression was detected mainly in the Stage 1 and Stage 2 populations in both CD4^−^ and CD4^+^ fractions (Figure 2C), whereas CD122 expression was mainly in the Stage 3 population as previously reported [21], and is inversely correlated with the expression of IL-17RB (Figure 2C). In order to investigate whether IL-17RB^+^ *i*NKT cells are distinct from IL-15-dependent *i*NKT cells, thymic *i*NKT cells from B6 and *Il15* ^L117P^ mice were divided based on the expression of IL-17RB and CD4, and were further analyzed in the expression of CD44 and NK1.1 (Figure 2D and 2E). The percentage of CD4^−^ and CD4^+^, IL-17RB^+^ *i*NKT cells was higher in *Il15* ^L117P^ mice (Figure 2D), due to the reduction in the numbers of IL-17RB^−^ *i*NKT cells. Concerning the distribution of the expression of CD44 and NK1.1 in *i*NKT cell subtypes, even though IL-17RB^+^ *i*NKT cells comprised only ∼10% of the thymic *i*NKT cells, more than half of them were Stage 2, while almost all (>97%) of the CD4^−^ and CD4^+^, IL-17RB^−^ *i*NKT cells were Stage 3 (Figure 2E). Furthermore, more than 80% of Stage 1/2 *i*NKT cells were IL-17RB^+^ *i*NKT cells, while only ∼2% of the Stage 3 *i*NKT cells were IL-17RB^+^ (Figure 2D). The percentage (Figure 2F) and absolute number (Figure 2G) of IL-17RB^+^ *i*NKT cells among the total *i*NKT cells and in developmental Stages 1 and 2 were similar to those of *Il15* ^L117P^ mice, while those of IL-17RB^−^ *i*NKT cells (i.e., CD122^+^ *i*NKT cells) among the total and in developmental Stage 3 were also comparable to those in *Il17rb* ^−/−^ mice, indicating that two distinct *i*NKT cell subsets are present in the different stages of *i*NKT cell development, i.e., the IL-17RB^+^ subtype in Stages 1 and 2 and the CD122^+^ subtype in Stage 3.

In order to determine if *i*NKT cell subtypes arise as a distinct population in the thymus of each other, each subtype in Stage 1 or Stage 2 was sorted and co-cultured with a fetal thymus (FT) lobe from *J*α*18* ^−/−^ mice (Figure 2H and 2I). IL-17RB^−^ subtype in Stage 1 gave rise to cells in Stage 2 and Stage 3 with IL-17RB^−^ phenotype (Figure 2H and 2I, lower left), whereas IL-17RB^+^ subtype in Stage 1 gave rise to cells in Stage 2 but not to Stage 3 with IL-17RB^+^ phenotype (Figure 2H and 2I, upper left). Furthermore, IL-17RB^−^ subtype in Stage 2 gave rise to cells in Stage 3 with IL-17RB^−^ phenotype (Figure 2H and 2I, lower left), whereas IL-17RB^+^ subtype in Stage 2 kept in Stage 2 with IL-17RB^+^ phenotype (Figure 2H and 2I, upper left), indicating that IL-17RB^+^ *i*NKT cells arise in the thymus as distinct phenotypic subtypes from IL-17RB^−^ *i*NKT cells, which undergo a series of developmental stages (i.e. Stages 1–3) previously characterized [14],[15].

To confirm the differences among subtypes of *i*NKT cells, we compared global gene expression profiles in WT B6 CD4^−^ or CD4^+^, IL-17RB^+^ or IL-17RB^−^, *i*NKT cells to each other (Figure S2A), and also WT B6 CD4^−^ or CD4^+^, IL-17RB^+^ *i*NKT cells to the same cell types from *Il15* ^L117P^ mice (Figure S2B). The genome-wide expression profile of the CD4^−^ and CD4^+^, IL-17RB^+^ *i*NKT cells were similar to each other but different from those of CD4^−^ and CD4^+^, IL-17RB^−^ *i*NKT cells (Figure S2A). Moreover, the gene expression profiles of CD4^−^ or CD4^+^, IL-17RB^+^ WT *i*NKT cells were similar to those in *Il15* ^L117P^ mice (Figure S2B). Therefore, it is likely that IL-17RB^+^ *i*NKT cell development in the thymus is distinct from the IL-17RB^−^ (i.e. CD122^+^) *i*NKT cells. The gene expression profiles of the CD4^+^ IL-17RB^+^ *i*NKT cells were quite similar to those of the CD4^−^ IL-17RB^+^ cells rather than the CD4^−^ or CD4^+^, IL-17RB^−^ *i*NKT cells (Figure S2A), suggesting that these two subtypes, CD4^−^ and CD4^+^, IL-17RB^+^ *i*NKT cells, develop from the same precursors, whereas the precursors for IL-17RB^−^ *i*NKT cells are distinct.

In order to investigate functional differences in the IL-17RB^+^ and IL-17RB^−^ subsets of *i*NKT cells, we analyzed the ability of thymic *i*NKT cells in B6, *Il17rb* ^−/−^ and *Il15* ^L117P^ mice to produce cytokines in response to α-GalCer (Figure S3). IFN-γ was produced at similar levels by *Il17rb* ^−/−^ and WT *i*NKT cells, but was greatly reduced in the *Il15* ^L117P^ *i*NKT cells, while the production of IL-9, IL-10, IL-13, IL-17A, and IL-22 was impaired in the *Il17rb* ^−/−^ but not in the *Il15* ^L117P^ *i*NKT cells, similar to what we had observed in the spleen and liver (Figure S3).

### Dominant Development of IL-17RB^+^ *i*NKT Subtypes in T_H_2-Prone BALB/c Mice

In a previous study, IL-17RB^+^ *i*NKT cells were fairly abundant in the spleen of T_H_2-prone mice, but were barely detectable in T_H_1-prone mice [17]. Thus, we examined whether the frequency of IL-17RB^+^ *i*NKT cells in the thymus of BALB/c mice is different from that of B6 mice. Intriguingly, more than one-third of thymic *i*NKT cells were IL-17RB^+^ in T_H_2-prone BALB/c mice, four times higher than in T_H_1-prone B6 mice (Figure S4A). The genome-wide expression profiles of CD4^−^ or CD4^+^, IL-17RB^+^ *i*NKT cells in BALB/c were similar to each other, but different from those of CD4^−^ or CD4^+^, IL-17RB^−^ *i*NKT cells (Figure S4B). Cluster analysis also showed that CD4^−^ or CD4^+^, IL-17RB^+^ or IL-17RB^−^ *i*NKT cells in B6 and BALB/c mice were essentially equivalent (Figure S4C).

### Genetic Analysis of *i*NKT Cell Subtypes in the Thymus

**Figure 3:**
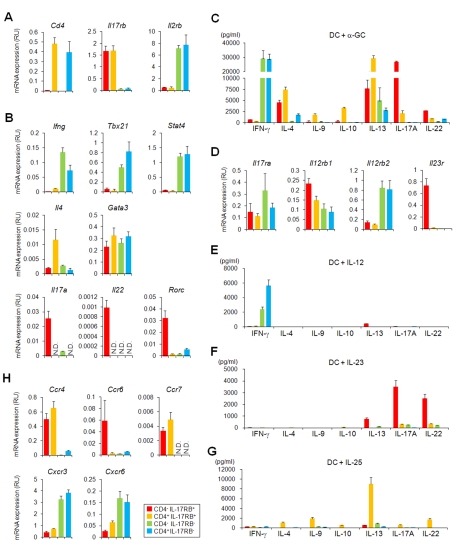
Differential gene expression and cytokine production among thymic *i*NKT cell subtypes from B6 mice. (A, B, D, H) Quantitative PCR analysis of thymic *i*NKT subtypes. Thymic *i*NKT cells further divided into four subtypes based on the expression of CD4 and IL-17RB (red, CD4^−^ IL-17RB^+^; orange, CD4^+^ IL-17RB^+^; blue, CD4^−^ IL-17RB^−^; green, CD4^+^ IL-17RB^−^). One representative out of three experiments is shown (mean ± SEM). (A) The purity of the sorted cells was confirmed by the relative *Il17rb* and *Cd4* mRNA expression levels in the respective subtypes. *Il2rb* ( = *Cd122*) expression was restricted to CD4^−^ and CD4^+^, IL-17RB^−^ *i*NKT cells. (B) Expression of T_H_1/T_H_2/T_H_17 related genes. T_H_1 related: *Ifng*, *Tbx21* and *Stat4*, T_H_2 related; *Il4* and *Gata3*, and T_H_17 related: *Il17a*, *Il22* and *Rorc* transcripts were analyzed. (D) Expression of cytokine receptor genes. Receptor for IL-12, IL-23, and IL-25 were analyzed. The component chains of the various receptors are IL-12 receptor: IL-12Rβ2/IL-12Rβ1; IL-23 receptor: IL-23R/IL-12Rβ1; IL-25 receptor: IL-17RB/IL-17RA. (H) Expression of chemokine receptor genes. *Ccr4*, *Ccr6*, *Ccr7*, *Cxcr3*, and *Cxcr6*. (C, E, F, G) In vitro cytokine production by thymic *i*NKT cell subtypes (red, CD4^−^ IL-17RB^+^; orange, CD4^+^ IL-17RB^+^; blue, CD4^−^ IL-17RB^−^; green, CD4^+^ IL-17RB^−^). Sorted thymic *i*NKT subtypes (5×10^4^ cells/100 µL) were co-cultured with BM-DCs (5×10^3^/100 µL) for 48 h in the presence of α-GalCer (100 ng/µL) (C), IL-12 (10 ng/µL) (E), IL-23 (10 ng/µL) (F), or IL-25 (10 ng/µL) (G). Levels of IFN-γ, IL-4, IL-9, IL-10, IL-13, IL-17A, and IL-22 were analyzed. The data are representative of three independent experiments (mean ± SEM).

*i*NKT cells in the thymus can be divided into four populations based on their expression of CD4 and IL-17RB (Figures 2D and S4A), and thymic *Il17rb* ^−/−^ *i*NKT cells had a decreased ability to produce T_H_2 and T_H_17 cytokines (Figure S3). Therefore, we analyzed the function of *i*NKT cell subtypes in the thymus of B6 (Figure 3) and BALB/c mice (Figure S5). We first used quantitative real-time PCR to investigate T_H_1/T_H_2/T_H_17-related gene expression patterns in FACS sorted thymic *i*NKT subtypes. The levels of *Cd4* and *Il17rb* transcripts were correlated with the surface expression of these molecules (Figures 3A and S5A). *Il2rb* ( = *Cd122*) expression was restricted to CD4^−^ and CD4^+^, IL-17RB^−^ *i*NKT cell subtypes (Figures 3A and S5A) in correlation with their surface protein expression (Figure 2C). The expression levels of T_H_1-related transcripts, such as *Ifng*, *Tbx21*, and *Stat4*, were more than 10 times higher in those of CD4^−^ and CD4^+^ IL-17RB^−^ *i*NKT cells. Higher levels of T_H_2-related transcripts, such as *Il4*, were detected in CD4^+^ IL-17RB^+^ *i*NKT cells, even though *Gata3*, a transcription factor essential for T_H_2 cytokine production, was expressed at a similar level in all subtypes (Figures 3B and S5B). On the other hand, the expression of T_H_17-related transcripts, such as *Il17a*, *Il22*, and *Rorc*, were restricted to the CD4^−^ IL-17RB^+^ *i*NKT cells (Figures 3B and S5B).

We then investigated the gene expression level in cells derived from Stages 1 and 2 by FT organ culture (Figure 2H and 2I). Consistent with the findings above, *Ifng* expression was restricted to the cells derived from CD4^−^ and CD4^+^, IL-17RB^−^ *i*NKT precursors (Figure S6A and S6B). Higher levels of *Il4* were detected in CD4^+^ IL-17RB^+^ derived cells and restricted expression of *Il17a* in cells derived from CD4^−^ IL-17RB^+^ precursors (Figure S6A and S6B), supporting each subtype arise from Stage 1 as a functionally distinct subtype.

Based on these findings, we analyzed potential production of cytokines from these thymic *i*NKT cell subtypes. Sorted *i*NKT cell subtypes were stimulated with PMA plus ionomycin (Figure S7A). Similar to the cytokine expression, IFN-γ was exclusively produced by the CD4^−^ and CD4^+^, IL-17RB^−^ subtypes, while IL-10 and IL-13 were mainly produced by the CD4^+^ IL-17RB^+^ *i*NKT cells and IL-17A was produced predominantly by CD4^−^ IL-17RB^+^ *i*NKT cells. It should be noted that all four subtypes have a potential to produce IL-4, in correlation with their mRNA expression of *Il4* and *Gata3* (Figures 3B and S5B).

We then further analyzed cytokine production after α-GalCer activation. Sorted *i*NKT cell subtypes were co-cultured with BM-DCs in the presence of α-GalCer (Figures 3C and S5C). IFN-γ was exclusively produced by the CD4^−^ and CD4^+^, IL-17RB^−^ subtypes, while IL-4, IL-9, IL-10, and IL-13 were mainly produced by the CD4^+^ IL-17RB^+^ *i*NKT cells. Similarly, IL-17A and IL-22 were produced predominantly by CD4^−^ IL-17RB^+^ *i*NKT cells. These cytokine production patterns correlated with their differential expression of T_H_1/T_H_2/T_H_17-related genes in the different *i*NKT subtypes.

We also analyzed the expression profiles of cytokine receptor genes. *Il12rb2* transcript was expressed in CD4^−^ and CD4^+^, IL-17RB^−^ *i*NKT cells, and *Il23r* expression was restricted to CD4^−^ IL-17RB^+^ *i*NKT cells (Figures 3D and S5D), suggesting that CD4^−^ and CD4^+^, IL-17RB^−^ *i*NKT cells respond to IL-12 through IL-12Rβ2/IL-12Rβ1, while CD4^−^ IL-17RB^+^ *i*NKT cells respond to IL-23 through IL-23R/IL-12Rβ1. In fact, CD4^−^ and CD4^+^, IL-17RB^−^ *i*NKT cells produced large amounts of IFN-γ but not T_H_2 and T_H_17 cytokines in response to IL-12 (Figures 3E and S5E), while CD4^−^ IL17RB^+^ *i*NKT cells produced large amounts of T_H_17 cytokines, IL-17A and IL-22, but not IFN-γ and T_H_2 cytokines in response to IL-23 (Figures 3F and S5F). IL-25-mediated activity requires not only IL-17RB but also IL-17RA expression [26], which is expressed on all *i*NKT cell subtypes (Figures 3D and S5D). IL-25 acts on thymic CD4^+^ IL-17RB^+^ *i*NKT cells to induce a large amount of T_H_2 cytokines, along with moderate amounts of T_H_17 cytokines (Figures 3G and S5G) similar to previous observations in the CD4^+^ IL-17RB^+^ *i*NKT cell subtype in the spleen [17]. Interestingly, however, IL-25 does not stimulate CD4^−^ IL-17RB^+^ *i*NKT cells, despite their expression of IL-17RB (Figures 3G and S5G). We also found that cytokine production from *i*NKT cells in these experimental settings was hardly observed when BM-DCs derived from *Cd1d1* ^−/−^ mice (unpublished data), indicating signals from TCR are also required for cytokine production from *i*NKT cells. These results suggest that three types of *i*NKT cells, i.e. CD4^−^ IL-17RB^+^ (*i*NKT-T_H_17, IL-23 reactive), CD4^+^ IL-17RB^+^ (*i*NKT-T_H_2/17, IL-25 reactive), and CD4^−^ and CD4^+^, IL-17RB^−^ (*i*NKT-T_H_1, IL-12 reactive), exist as distinct subpopulations in the thymus.

The chemokine receptor expression patterns are also distinct among thymic *i*NKT cell subtypes. *Ccr4* and *Ccr7* expression was restricted to both CD4^−^ and CD4^+^, IL-17RB^+^ *i*NKT cells, and *Ccr6* expression was only observed on CD4^−^ IL-17RB^+^ *i*NKT cells (Figures 3H and S5H). *Cxcr3* expression was several times higher on IL-17RB^−^ *i*NKT cells than on the other subtypes. Surprisingly, the expression of *Cxcr6*, which has been reported to be abundantly expressed by all *i*NKT cells [27],[28], was also restricted to the IL-17RB^−^ *i*NKT cells (Figures 3H and S5H). Note that the expression patterns and levels of all of the genes tested were almost equivalent between B6 and BALB/c mice, consistent with our finding that all *i*NKT subtypes are present in these strains.

### Distribution of *i*NKT Subtypes in the Periphery

**Figure 4:**
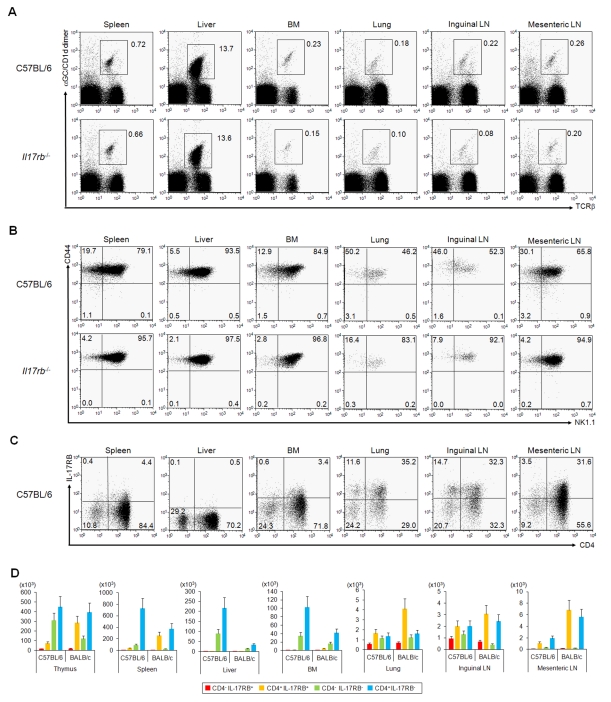
*i*NKT cell subtypes in the periphery. (A–C) FACS profile of peripheral *i*NKT cells in B6 mice. α-GalCer/CD1d dimer^+^ TCRβ^+^ *i*NKT cells (A) and *i*NKT subtypes based on the expression of CD44 and NK1.1 (B) or CD4 and IL-17RB (C) in spleen, liver, BM, lung, inguinal LN, and mesenteric LN from B6 or *Il17rb* ^−/−^ mice. Numbers indicate percentage of total mononuclear cells (A) and *i*NKT cells (B, C). (D) Number of cells of each *i*NKT subtype based on the expression of IL-17RB and CD4 in thymus and periphery of B6 and BALB/c mice. Cell numbers were calculated based on the results from Figures 4A, 4C, S8A, and S8B. IL-17RB^+^ *i*NKT cells were mainly localized in spleen, lung, inguinal LN, and mesenteric LN, whereas hardly any were observed in liver and BM. One representative experiment of three is shown.

Distinct expression of chemokine receptors among thymic *i*NKT cell subtypes (Figures 3H and S5H) may reflect the differential distribution of *i*NKT cell subtypes in the periphery. We thus investigated the frequency of total *i*NKT cells and subtypes in the spleen, liver, BM, lung, inguinal lymph node (LN), and mesenteric LN in WT B6, BALB/c, and *Il17rb* ^−/−^ mice (Figures 4 and S8). The absolute number and percentage of *i*NKT cells were slightly decreased in the spleen, lung, inguinal LN, and mesenteric LN of *Il17rb* ^−/−^ mice, but were unchanged compared to WT mice in liver and BMs (Figures 4A and S8A). We then gated on α-GalCer/CD1d dimer^+^ TCRβ^+^ *i*NKT cells and further analyzed them for the expression of CD44 and NK1.1 in B6 background mice (Figure 4B). The percentage of NK1.1^−^ subtype cells was higher in the spleen, lung, inguinal LN, and mesenteric LN, but lower in the liver and BM, and was decreased in *Il17rb* ^−/−^ mice, suggesting that the majority of *i*NKT cell subtypes maintain surface expression of NK1.1^−^ after emigration from the thymus (Figure 2B). Similarly, we examined the expression of CD4 and IL-17RB on the *i*NKT subtypes (Figures 4C and S8B). Interestingly, IL-17RB^+^ *i*NKT cells were abundant in the lung, inguinal LN, and mesenteric LN, but barely detectable in the liver and BM of both B6 and BALB/c mice. More than 40% of *i*NKT cells were IL-17RB^+^ in the lung, inguinal LN, and mesenteric LN, whereas more than 90% were IL-17RB^−^ in the liver and BM (Figures 4C and S8B). Therefore, the distribution patterns of the *i*NKT cell subtypes are distinct in the tissues. In agreement with a previous study [21], we found that the number of *i*NKT cells was decreased in the spleen (∼1/3) and liver (∼1/30) in *Il15* ^L117P^ mice (Figure S9A). Reduction of *i*NKT cell number was also observed in BM (∼1/8) in these mice (Figure S9A), probably due to the selective reduction of the IL-17RB^−^ subtypes (Figure S9B). We finally compared *i*NKT cell subtypes in the thymus and periphery of B6 and BALB/c mice (Figure 4D). The total *i*NKT cell number was almost equivalent between these two strains, but BALB/c had ∼4 times more CD4^+^ IL-17RB^+^ subtype cells, but lower (∼1/3) numbers of CD4^−^ IL-17RB^−^ cells, resulting in a higher number of CD4^+^ IL-17RB^+^ cells in the spleen (∼5 times), lung (∼2 times), inguinal LN (∼1.5 times), mesenteric LN (∼4 times), and lower numbers of CD4^−^ IL-17RB^−^ cells, especially in liver (∼1/6) and BM (∼2/5) of BALB/c mice (Figure 4D).

To confirm the distribution profiles of each subtype in the periphery, we performed intracellular cytokine staining after PMA plus ionomycin stimulation (Figure S7B) and quantitative real-time PCR analysis (Figure S10A–D) on these *i*NKT cells that were tested in the thymic *i*NKT cell subtypes (Figures 3A, 3B, 3D, 3H, S7A). The gene expression profiles and potential cytokine production in the *i*NKT cell subtypes were almost equivalent among those in the different peripheral tissues, but higher than those in the thymus, strongly suggesting that each *i*NKT subtype in the periphery is derived from the same *i*NKT subtypes in the thymus.

### IL-17RB^+^ *i*NKT Cells as T_H_2/T_H_17-Producing *i*NKT Subtypes

**Figure 5:**
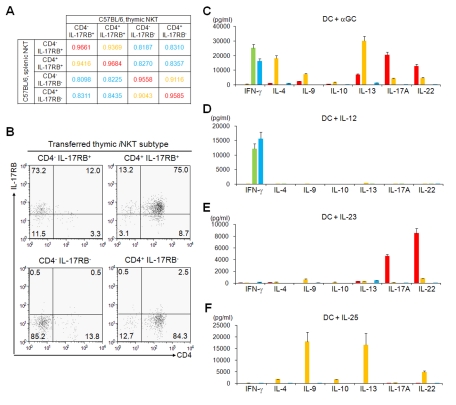
Function of *i*NKT cell subtypes in the spleen. (A) Global gene expression profiles in *i*NKT subtypes in the thymus and spleen. Tree view representation of clustering analysis among the four *i*NKT subtypes in thymus and spleen from B6 and BALB/c. The values represent coefficients between the indicated panels. *r* ^2^>0.95 in red, 0.85<*r* ^2^<0.95 in orange, and *r* ^2^<0.85 in blue. One representative experiment of three is shown. (B) Plasticity and stability of *i*NKT subtypes. The four *i*NKT cell subtypes in the thymus were sorted and each subtype (5×10^5^) was i.v. transferred into independent *Jα18* ^−/−^ mice (*n* = 3). 10 d after transfer, α-GalCer/CD1d dimer^+^ TCRβ^+^ cells in spleen were analyzed by FACS for the expression of IL-17RB and CD4. Representative data from three experiments are shown. (C–F) In vitro cytokine production by splenic *i*NKT cell subtypes (red, CD4^−^ IL-17RB^+^; orange, CD4^+^ IL-17RB^+^; blue, CD4^−^ IL-17RB^−^; green, CD4^+^ IL-17RB^−^). Sorted splenic *i*NKT subtypes (5×10^4^ cells/100 µL) were co-cultured with BM-DCs (5×10^3^/100 µL) for 48 h in the presence of α-GalCer (100 ng/µL) (C), IL-12 (10 ng/µL) (D), IL-23 (10 ng/µL) (E), and IL-25 (10 ng/µL) (F). Levels of IFN-γ, IL-4, IL-9, IL-10, IL-13, IL-17A, and IL-22 in the supernatants were analyzed by ELISA or CBA. Data are mean ± SD of triplicate wells. One representative experiment of three is shown.

We next compared global gene expression profiles of CD4^−^ or CD4^+^, IL-17RB^+^ or IL-17RB^−^ *i*NKT subtypes in the thymus and spleen in order to test whether each subtype is functionally and phenotypically stable or plastic. Each of the four subtypes in spleen was highly correlated with the corresponding subtype in the thymus (Figure 5A), suggesting that *i*NKT subtypes can be divided by CD4 and IL-17RB expression both in the thymus and the periphery. Furthermore, *i*NKT cell subtypes in the periphery (Figure S10) showed similar quantitative gene expression profiles as in the thymus (Figure 3).

In order to confirm the stability and plasticity of *i*NKT cell subtypes, we sorted thymic *i*NKT cell subtypes based on the expression of CD4 and IL-17RB from WT B6 and transferred them into *i*NKT cell-deficient *J*α*18* ^−/−^ mice. Ten days after transfer, we analyzed the IL-17RB expression by *i*NKT cell subtypes in the spleen. The results clearly showed that the majority of transferred cells maintained their surface IL-17RB expression (Figure 5B), suggesting that IL-17RB expression is stable as the cells migrate from the thymus to the periphery.

We further analyzed in cytokine production of splenic *i*NKT cell subtypes from B6 and BALB/c mice. The cytokine production profiles of splenic *i*NKT cell subtypes in response to α-GalCer (Figures 5C and S11A), IL-12 (Figures 5D and S11B), IL-23 (Figures 5E and S11C), and IL-25 (Figures 5F and S11D) were quite similar to those of the thymic *i*NKT cell subtypes (Figures 3C, 3E, 3F, 3G, S5C, S5E, S5F, S5G). Taken together, all of the *i*NKT subtypes detected in the thymus also exist as phenotypically and functionally distinct subtypes in the peripheral tissues.

### E4BP4 Is Required for the Production of IL-9, IL-10, IL-13, IL-17A, and IL-22 Cytokines by CD4^+^ IL-17RB^+^ *i*NKT Cells in Response to IL-25

Both thymic and peripheral *i*NKT cells in the steady state contain *Ifng* mRNA in the CD4^−^ and CD4^+^, IL-17RB^−^ cells (*Tbx21* expressed, *i*NKT-T_H_1, IL-12 reactive), and *Il17a* and *Il22* mRNA in the CD4^−^ IL-17RB^+^ cells (*Rorc* expressed, *i*NKT-T_H_17, IL-23 reactive). The expression of these cytokine transcripts is thought to result from the fact that peripheral *i*NKT cells are not truly quiescent, but instead appear to be continuously activated at a low level due to their recognition of endogenous self-glycolipid ligand(s) in vivo. However, the CD4^+^ IL-17RB^+^ *i*NKT cells do not contain *Il9*, *Il10*, *Il13* (unpublished data), *Il17a*, or *Il22* mRNA (Figures 3B, S5B, S10B) in the steady state, even though these cytokines are immediately produced after activation by α-GalCer, similar to cases of IFN-γ from IL-17RB^−^ *i*NKT cells. These results suggest differences in the transcriptional regulation of cytokine genes in the different *i*NKT cell subtypes.

One of the candidate genes is E4BP4, a mammalian basic leucine zipper transcription factor that regulates IL-10 and IL-13 production not only by CD4^+^ T cells and regulatory T cells but also by *i*NKT cells [29]. E4BP4 expression was markedly induced in IL-25-treated *i*NKT cells, and its expression level correlated with *Il10* and *Il13* expression [29]. Furthermore, *i*NKT cells lacking *E4bp4* had reduced expression of IL-10 and IL-13 in response to either IL-25 or α-GalCer stimulation, but the IFN-γ and IL-4 production were unaffected [29], indicating that *E4bp4* controls the T_H_2 cytokine production in a particular *i*NKT cell subtype.

**Figure 6:**
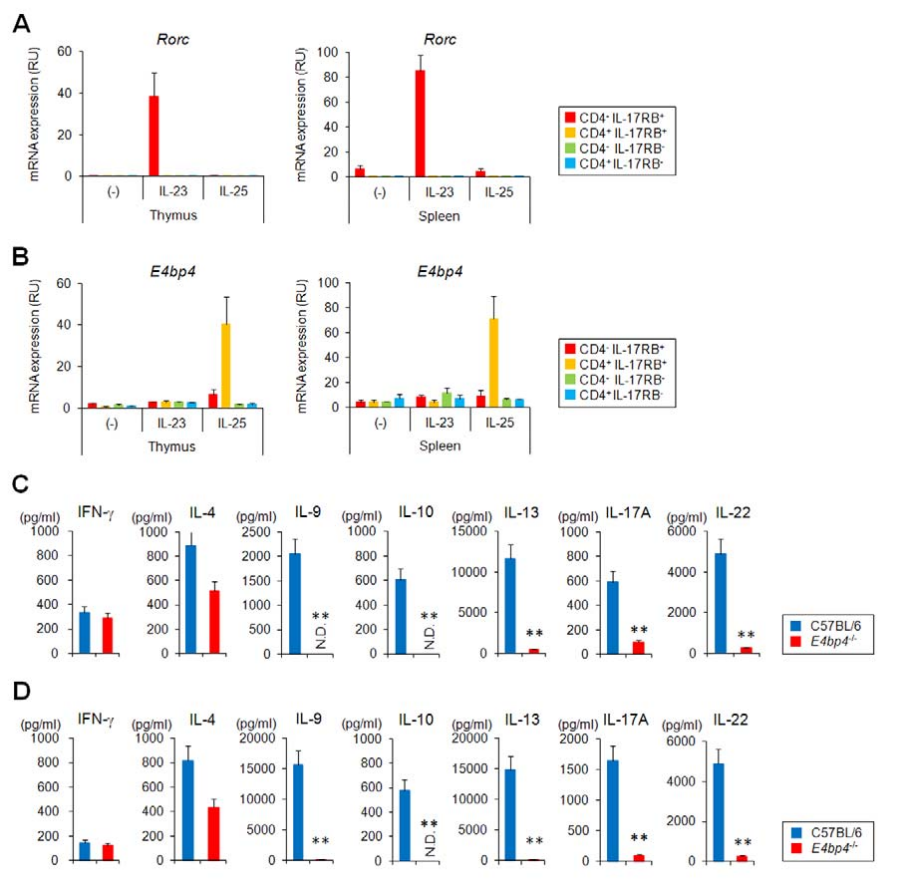
Involvement of *E4bp4* in cytokine production by CD4^+^ IL-17RB^+^ *i*NKT cells in response to IL-25. (A, B) Quantitative analysis of *Rorc* (A) and *E4bp4* (B) in *i*NKT cell subtypes after cytokine treatment. Sorted *i*NKT cell subtypes (5×10^4^/100 µL) from thymus (left) or spleen (right) were co-cultured with BM-DCs (5×10^3^/100 µL) in the presence or absence of IL-23 (10 ng/ml) or IL-25 (10 ng/ml) for 24 h. The *i*NKT cell subtypes were then sorted again and analyzed for expression of the indicated genes by quantitative real-time PCR. The data are representative of three independent experiments (mean ± SEM). (C, D) Cytokine production by CD4^+^ IL-17RB^+^ *i*NKT cells in response to IL-25. Sorted CD4^+^ IL-17RB^+^ *i*NKT cells (5×10^4^/100 µL) from thymus (C) or spleen (D) of B6 or *E4bp4* ^−/−^ mice were co-cultured with BM-DCs (5×10^3^/100 µL) in the presence of IL-25 (10 ng/ml) for 48 h and then the levels of the indicated cytokines in the tissue culture media were analyzed. *i*NKT cells from B6 were compared to those from *E4bp4* ^−/−^ mice. ** *p*<0.01 calculated by *t* test. The data are representative of three independent experiments (mean ± SD).

Therefore, we analyzed the role of *E4bp4* in *i*NKT cell subtypes. The expression of *E4bp4* was selectively and strongly induced by IL-25 treatment in CD4^+^ IL-17RB^+^ *i*NKT cells both from thymus and spleen (Figure 6B). However, CD4^−^ IL-17RB^+^ *i*NKT cells failed to induce *E4bp4* expression even after treatment with IL-23 (Figure 6B), suggesting the cell type-specific function of *E4bp4* and its possible role not only in *Il10* and *Il13* expression but also in *Il9*, *Il17a*, and *Il22* expression by IL-25-treated CD4^+^ IL-17RB^+^ *i*NKT cells. To test this hypothesis, we analyzed cytokine production by CD4^+^ IL-17RB^+^ *i*NKT cells lacking *E4bp4* after treatment with IL-25 in the presence of BM-DCs (Figure 6C). The production of IL-9, IL-10, IL-13, IL-17A, and IL-22 cytokines by both thymic or splenic CD4^+^ IL-17RB^+^ *i*NKT cells in response to IL-25 was completely abrogated, indicating E4BP4 turned out to be an intrinsic regulator of IL-25-mediated production, not only of IL-10 and IL-13 but also of IL-9, IL-17A, and IL-22.

### Involvement of IL-17RB^+^ *i*NKT Cells in the Pathogenesis of Virus-Induced AHR

We then investigated the role of IL-17RB^+^ *i*NKT cells in the pathogenesis of virus-induced AHR, which is known to be different from allergen-induced AHR [30]. Certain viruses, such as respiratory syncytial virus (RSV), Sendai virus, metapneumovirus, and parainfluenza virus, cause childhood asthma and COPD-like symptoms, which include AHR, airway inflammation, and mucus hypersecretion [31]–[33]. However, it has been very difficult to understand how such symptoms develop, even long after the apparent clearance of viruses. It has been reported that, in mouse models of infection with parainfluenza virus or Sendai virus, virus-induced chronic inflammation leads to asthma that resembles human asthma and COPD [34]. The chronic pulmonary symptoms evolved independently of CD4^+^ T cells but required CD4^−^ *i*NKT cells and did not occur in *Cd1d* ^−/−^ and *J*α*18* ^−/−^ mice [34].

**Figure 7:**
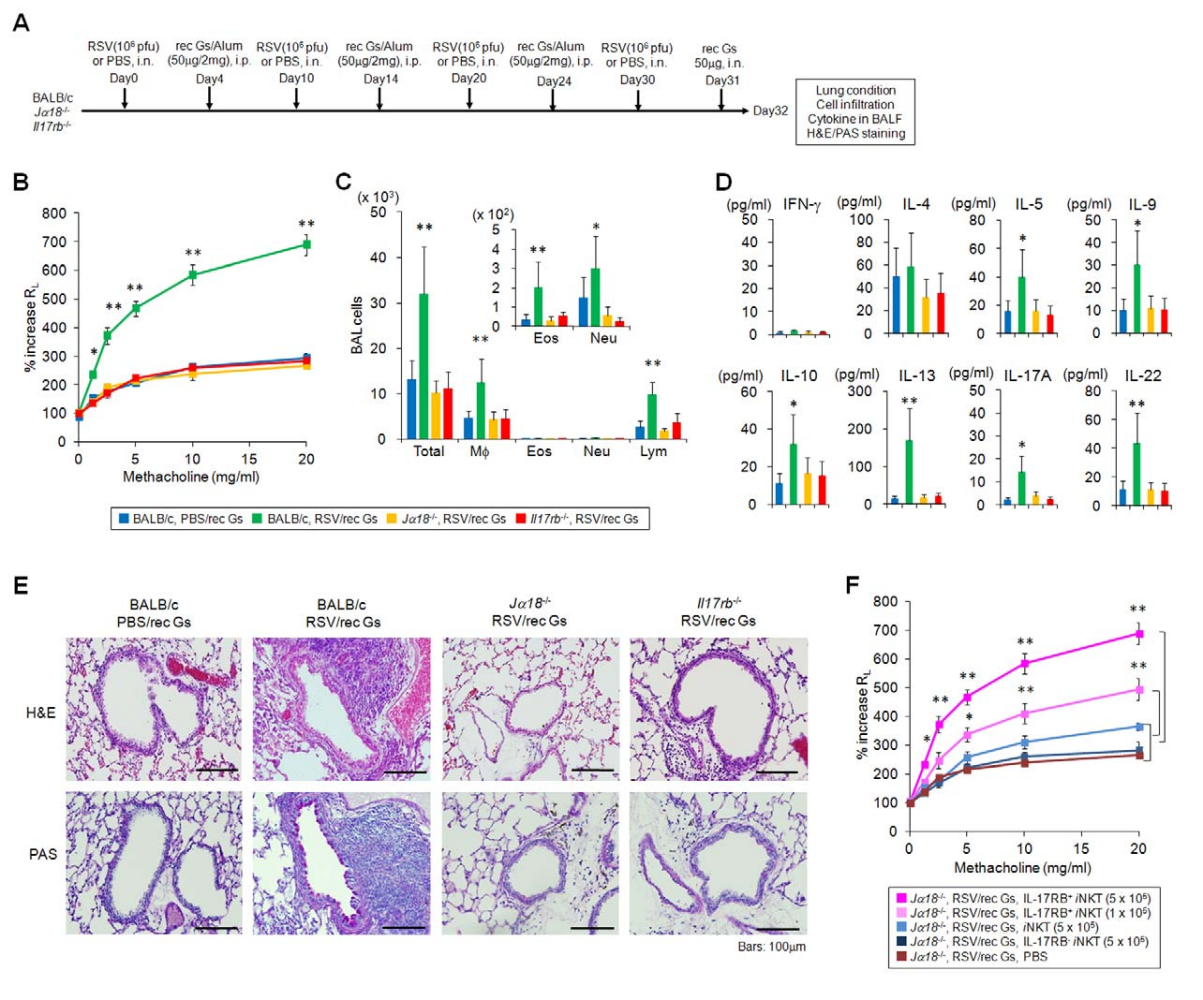
Involvement of IL-17RB^+^ *i*NKT cells in the development of RSV-induced AHR. (A) Schematic showing the protocol for RSV-induced AHR. Mice were i.n. administered with RSV (10^6^ pfu) or PBS alone as a control 4 times at 10-day intervals. Mice were i.p. immunized with rec Gs/alum (50 µg/2 mg) 4 d after first RSV infections. Three days after the last RSV administration, mice were exposed i.n. to rec Gs and were measured 1 d later. (B) Development of RSV-induced AHR in BALB/c, but not in *Jα18* ^−/−^ or *Il17rb* ^−/−^ mice. Changes in R_L_ are depicted. The RSV-infected, rec Gs immunized, BALB/c mice had a greatly increased AHR compared to the other three groups. Results are expressed as the mean ± SEM. * *p*<0.05 and ** *p*<0.01. (C, D) Total and differential cell counts (C) and cytokines (D) in BAL fluid. BAL fluid was collected 24 h after challenge of the mice depicted in (B) with intranasal rec Gs. Results are expressed as the mean ± SEM. * *p*<0.05 and ** *p*<0.01. (E) Histological examination of lung tissues by H&E and PAS staining. RSV infected, rec Gs immunized, BALB/c, *Jα18* ^−/−^ or *IL17rb* ^−/−^, mice were compared with control BALB/c mice (rec Gs alone). Bars indicate 100 µm. (F) AHR development after cell transfer of spleen IL-17RB^+^ *i*NKT cells into *Jα18* ^−/−^ mice. Indicated cell numbers of sorted IL-17RB^+^, IL-17RB^−^ *i*NKT cells or total *i*NKT cells from spleen, or PBS control, were i.v. transferred into rec-Gs/alum-sensitized *Jα18* ^−/−^ mice 24 h before RSV treatment (on the day 9, 19, and 29), and then challenged with rec Gs (24 h) and measurement of lung resistance (48 h). Each group of IL-17RB^+^ *i*NKT cell-transferred mice was compared to other three groups. * *p*<0.05, ** *p*<0.01 calculated by Kruskal Wallis test. The results represent one out of four experiments with five mice in each group.

Therefore, we attempted to determine whether or not the CD4^−^ IL-17RB^+^ *i*NKT cells are responsible for chronic inflammatory lung disease induced by RSV infection. We used the secreted form of recombinant G protein of RSV (rec Gs) (Figure S12) as an immunogen because priming with a recombinant vaccinia virus (rVV) expressing rec Gs induced a more T_H_2-biased response and enhanced pulmonary eosinophil and macrophage infiltration following RSV challenge than did priming with rVV expressing either wild-type G or membrane anchored G (Gm) proteins [35],[36]. Mice were inoculated i.n. with RSV (10^6^ pfu/100 µl) or PBS as a control four times at 10-d intervals and were intraperitoneally (i.p.) immunized with rec Gs/alum (50 µg/2 mg) 4 d after the first RSV infection. Three days after the last RSV administration, mice were exposed i.n. to 50 µg rec Gs and then, 24 h later, measured for AHR (Figure 7A). In this experimental setting, RSV/rec Gs-induced AHR was observed in WT BALB/c but not in *Jα18* ^−/−^ or *Il17rb* ^−/−^ mice, which had a similar response level as PBS/rec Gs-induced WT controls, indicating that IL-17RB^+^ *i*NKT cells contribute to the development of RSV plus viral antigen-induced AHR (Figure 7B). Airway macrophage and lymphocyte numbers, which were relatively higher than eosinophils and neutrophils, were recruited into the bronchoalveolar lavage (BAL) fluid of RSV/rec Gs-induced WT mice but not the other mice (Figure 7C). These results suggest that IL-17RB^+^ *i*NKT cells are required for the development of RSV-induced AHR. Low level of cytokines (IL-4, IL-9, IL-10, IL-13, IL-17A, and IL-22) in the BAL fluid was detected in this experiment (Figure 7D). The production of IL-13 and IL-22, which plays a crucial role in the activation of macrophages and neutrophils, respectively, was detected higher in RSV/rec Gs-induced WT mice. Hematoxylin and eosin (H&E) staining of the lung tissue revealed that a large number of inflammatory mononuclear cells had infiltrated into the peribronchiolar region, a response that was higher in RSV/rec Gs-induced WT mice compared to RSV/rec Gs-induced *Jα18* ^−/−^ or *Il17rb* ^−/−^, mice (Figure 7E, upper panel). By periodic acid-Schiff (PAS) staining, mucus-producing cells were abundant only in RSV/rec Gs-induced BALB/c mice but not in *J*α*18* ^−/−^ or *Il17rb* ^−/−^ mice (Figure 7E, lower panel). To confirm the findings that IL-17RB^+^ *i*NKT cells are essential for the development of RSV/rec Gs-induced AHR, we transferred enriched splenic IL-17RB^+^ *i*NKT cells into *Jα18* ^−/−^ mice and tested their ability to develop AHR (Figure 7F). The cell transfer of IL-17RB^+^ *i*NKT cells, but not IL-17RB^−^ *i*NKT cells nor PBS alone, restored AHR induced by RSV plus rec Gs, dependent of cell number transferred, demonstrating the important contribution of IL-17RB^+^ *i*NKT cells in the pathogenesis of development in virus plus viral antigen-induced AHR.

## Discussion

In the present study, we identified IL-17RB^−^ and IL-17RB^+^ subtypes of *i*NKT cells both in the thymus and the periphery. The IL-17RB^−^ *i*NKT cells express CD122 (IL-15Rβ chain), expand in an IL-15-dependent manner, and produce IFN-γ in response to IL-12. On the other hand, the IL-17RB^+^ *i*NKT cells do not express CD122 or respond to IL-15. The IL-17RB^+^ *i*NKT cells can be further divided into at least two subtypes: (1) CD4^+^ IL-17RB^+^ *i*NKT cells produce T_H_2, T_H_9, and T_H_17 cytokines in an E4BP4-dependent fashion in response to IL-25, and (2) CD4^−^ IL-17RB^+^ *i*NKT cells are RORγt^+^ and produce T_H_17 cytokines in response to IL-23, but independently of E4BP4. In the thymus, the IL-17RB^+^ *i*NKT cells have a developmental pathway distinct from the IL-17RB^−^ *i*NKT cells.

It has been proposed that *i*NKT cell differentiation stages can be categorized based on the expression patterns of CD44 and NK1.1, for example CD44^lo^ NK1.1^−^ for Stage 1, CD44^hi^ NK1.1^−^ for Stage 2, and CD44^hi^ NK1.1^+^ for Stage 3 [14],[25]. However, the majority (>80%) of IL-17RB^+^ *i*NKT cells was present in both the Stage 1 and Stage 2 subsets, while IL-17RB^−^ *i*NKT cells were enriched in *Il17rb* ^−/−^ mice and were mainly detected in Stage 3, suggesting that a certain but not all of the Stage 1 and Stage 2 IL-17RB^+^ *i*NKT cells are not precursors for the Stage 3 cells. It is believed that *i*NKT cells acquire their ability to produce IL-4 and IL-10, but make little IFN-γ in Stages 1/2 populations, whereas *i*NKT cells in Stage 3 produce abundant IFN-γ but less if any IL-10 [14],[15],[25],[37]. These finding are in agreement with the present results that the Stage 1/2 populations mainly contain IL-17RB^+^ *i*NKT cells that can produce IL-4 and IL-10, but not IFN-γ, whereas the majority of the Stage 3 *i*NKT cells are IL-17RB^−^ *i*NKT cells producing IFN-γ but not T_H_2 cytokines. The results shown here also indicated that all of the four *i*NKT subtypes already existed in Stage 1 and developed into phenotypically and functionally distinct *i*NKT cells as CD4^−^ or CD4^+^, IL-17RB^+^ in Stage 2 and CD4^−^ or CD4^+^, IL-17RB^−^ through Stage 2 to Stage 3.

It has reported that IL-15 plays an important role in the expansion of *i*NKT cells [21]. Our present data showed that IL-15 requires only for the expansion of IL-17RB^−^ *i*NKT cell subtypes but not for IL-17RB^+^ *i*NKT cells, even though it has still been unclear that the cytokine(s) are required for the development and expansion of IL-17RB^+^ subtypes. In fact, IL-17RB^−^ *i*NKT cell subtypes were greatly reduced in number among *i*NKT cell subtypes but already had an ability to produce IFN-γ in *Il15* ^L117P^ mice, resulting in the reduced IFN-γ production after *i*NKT cell activation due to the reduced number of these subtypes.

In the previous reports, the IL-17A-producing subtypes were proposed to be contained within the CD44^hi^ NK1.1^−^ CD4^−^ RORγt^+^ subpopulation [8],[19]. In the present studies, we found that the CD4^−^ IL-17RB^+^ *i*NKT cell subtype is CD44^hi^ NK1.1^−^ CD4^−^ (about 50%–70% of the cells are IL-17RB^+^) and has a restricted expression of *Il17a*, *Rorc*, *Ccr6*, and *Il23r* genes, for a phenotype similar to the previously reported CD44^+^ NK1.1^−^ CD4^−^ RORγt^+^ population that produces IL-17A [19],[38]. These results indicate that IL-17RB (and CD4^−^) is a reliable and specific phenotypic marker for RORγt^+^ IL-17A-producing *i*NKT cells in the thymus.

In the periphery, the tissue distribution of the *i*NKT cell subtypes seems to largely depend on the expression of chemokine receptors: CCR6^+^ CCR4^+^ CCR7^+^ expression by CD4^−^ IL-17RB^+^ *i*NKT cells, CCR4^+^ CCR7^+^ expression by CD4^+^ IL-17RB^+^ *i*NKT cells, and CXCR3^+^ CXCR6^+^ by CD4^−^ and CD4^+^, IL-17RB^−^ *i*NKT cells. Indeed, the number of liver *i*NKT cells, the majority of which are the CD4^−^ and CD4^+^, IL-17RB^−^ *i*NKT cells identified here, depends on the chemokine receptor CXCR6, whereas *i*NKT cells in other tissues are less dependent as reported [27],[28]. In *Ccr4* ^−/−^ mice, the lung has fewer *i*NKT cells and a corresponding reduction in *i*NKT cell-mediated AHR [39], implicating the reduction of pulmonary localization of IL-17RB^+^ *i*NKT cells.

IL-17A-producing *i*NKT cells have been described in other studies in the thymus, liver, spleen, lung, LNs, and skin [8],[19],[20],[38],[40]. In these studies, it was suggested that all NK1.1^−^ *i*NKT cells have the potential to secrete IL-17A. However, in the present study, we show heterogeneity among NK1.1^−^ *i*NKT cells. Accordingly, CD4^−^ but not CD4^+^, IL-17RB^+^ *i*NKT cells correspond to the IL-17A-producing *i*NKT cells previously reported, as does the exclusive expression of *Ccr6* along with *Itgae* ( = *Cd103*) and *Il1r1* ( = *Cd121a*) in CD4^−^ IL-17RB^+^ *i*NKT cells (unpublished data) [40].

CD4^+^ IL-17RB^+^ *i*NKT cells produce not only the previously described IL-13 and IL-4 [17],[18] but also IL-9 and IL-10 along with IL-17A and IL-22 in response to IL-25 in an E4BP4-dependent fashion. Even though it is still unclear whether IL-25-reactive CD4^+^ IL-17RB^+^ *i*NKT cells can be further divided into differentially functional subsets (i.e. *i*NKT-T_H_2, *i*NKT-T_H_9, *i*NKT-T_H_17), it is noteworthy that a recently described subset of differentiated T cells [41], termed T_H_9, which can be induced by IL-4 plus TGF-β, produces IL-9 and IL-10 in response to IL-25. This IL-9 production is IL-4 independent, highlighting the role of IL-25 in the regulation of both T_H_2 and T_H_9 cells [42]. We demonstrated here that IL-25 induces not only IL-13 and IL-4 but also IL-9 and IL-10 from CD4^+^ IL-17RB^+^ *i*NKT cells, which can thus be characterized as *i*NKT-T_H_2 and *i*NKT-T_H_9 cells. Concerning the cytokine production by CD4^+^ IL-17RB^+^ *i*NKT cells in response to IL-25, not only IL-10 and IL-13 but also IL-9, IL-17A, and IL-22 were attenuated in the absence of *E4bp4*, recently defined as a transcription factor that regulates IL-10 and IL-13 production by CD4^+^ T cells and *i*NKT cells [29], suggesting that E4BP4 also controls IL-25-mediated production of IL-9, IL-17A, and IL-22. Although the precise mechanisms by which IL-25 mediates cytokine expression still remains unclear, E4BP4 itself directly or indirectly controls IL-9, IL-10, IL-13, IL-17A, and IL-22 expression by genetic/epigenetic regulation in CD4^+^ IL-17RB^+^ *i*NKT subtypes. It will be of interest to determine if E4BP4 regulates IL-9, IL-17A, and IL-22 production by CD4^+^ T_H_ cells. Taken collectively, our studies indicate that CD4^−^ or CD4^+^, IL-17RB^+^ *i*NKT cells become functionally stable *i*NKT-T_H_17 or *i*NKT-T_H_2/9/17, respectively, during their development.

The study described here indicates that *i*NKT cell-mediated AHR was not induced by viral infections in *Jα18* ^−/−^ or *Il17rb* ^−/−^ mice, suggesting that IL-17RB^+^ *i*NKT cells are responsible for the pathogenesis of many different forms of airway inflammation. Although distinct subsets of *i*NKT cells have been reported to be involved in different forms of asthma [17],[18],[34],[43], they are now consolidated into CD4^−^ and/or CD4^+^ IL-17RB^+^ *i*NKT cell subsets.

*i*NKT cells are also known to mediate regulatory functions controlling various pathological conditions, such as infectious diseases caused by microbes [44], autoimmune diseases (colitis, lupus, diabetes) [45],[46], atherosclerosis [47], and malignancy [48]. It will be interesting to elucidate whether subsets of *i*NKT cells play differential roles in mediating and controlling these diverse pathological conditions.

## Materials and Methods

### Mice

B6 and BALB/c mice were purchased from Charles River Laboratories or Clea Japan, Inc. *Il17rb*-deficient mice were generated as shown in Figure S1 and were backcrossed >8 times to B6 or BALB/c mice. *Il15* ^L117P^ mutant mice were produced by *N*-Ethyl-*N*-nitrosourea (ENU) mutagenesis by ENU administration to male C57BL/6J mice, and their sperm was mated to wild-type eggs and preserved as founder embryos [49],[50]. *Jα18*-deficient mice were generated as previously described [51] and were backcrossed >10 times to B6 or BALB/c mice. *Cd1d1*-deficient mice [52] were provided by Dr. Luc van Kaer (Nashville, TN). *E4bp4*-deficient mice were generated as previously described and were backcrossed 8 times to B6 mice [29]. All mice were kept under specific pathogen-free conditions and were used at 8–16 wk of age. All experiments were in accordance with protocols approved by the RIKEN Animal Care and Use Committee.

### Cytokine Measurement

Cytokines except IL-22 in culture supernatants and BAL fluids were analyzed by cytometric bead array (BD Biosciences) according to the manufacturer's protocol. IL-22 was quantified by an ELISA reagent set (eBioscience) according to the manufacturer's protocol.

### Flow Cytometry and Cell Sorting

Cells were analyzed by FACS Calibur (BD Biosciences) or FACS Canto II (BD Biosciences) and sorted by FACS Aria (BD Biosciences). Antibodies (BD Biosciences or eBioscience) used for staining mouse cells were as follows: FITC or APC-Cy7 anti-TCRβ (H57-597), Pacific blue anti-CD4 (RM4-5), FITC anti-CD44 (IM7), PE-Cy7 anti-NK1.1 (PK136), PE anti-CD122 (TM-β1), FITC anti-CD8α (53-6.7), PerCP-Cy5.5 anti-CD25 (PC61), PE anti-IFN-γ (XMG1.2), PE anti-IL-4 (11B11), PE anti-IL-10 (JES5-16E3), PE anti-IL-13 (eBio13A), PE anti-IL-17A (TC11-18H10), and PE rat IgG1 (A110-1). Biotinylated anti-mouse IL-17RB (B5F6) was generated previously [17] and detected by staining with PE or PE-Cy7 Avidin (BD Biosciences). APC α-GalCer loaded CD1d dimer (BD Biosciences) for *i*NKT cell enrichment and detection was prepared as previously described [53].

### Coculture with a FT lobe

The procedures for the coculture with a deoxyguanosine (dGuo)-treated FT lobe under high oxygen submersion conditions have been described in detail previously [54],[55]. Basically, single dGuo-treated FT lobes from *Jα18* ^−/−^ of B6 background were placed into wells of a 96-well V-bottom plate, to which cells from B6 mice to be examined were added. Culture medium was supplemented with IL-7 (1 ng/ml), IL-15 (10 ng/ml), and soluble IL-15Rα (10 ng/ml). The plates were centrifuged at 150× g for 5 min at room temperature, placed into a plastic bag (Ohmi Odor Air Service), the air inside was replaced by a gas mixture (70% O_2_, 25% N_2_, and 5% CO_2_), and incubated at 37°C. After 10 d of culture, cells were harvested from each well and analyzed by FACS and quantitative real-time PCR.

### Intracellular Cytokine Staining

Intracellular cytokine staining was performed as described previously [53]. For cytokine production from sorted *i*NKT cell subtypes, Brefeldin A (Sigma-Aldrich) was added for the last 4 to 5 h of culture to accumulate intracellular cytokines after PMA (25 ng/ml, Sigma) with ionomycin (1 µg/ml, Sigma) treatment. Following fixation with Cytofix/Cytoperm plus (BD Biosciences), cells were stained for indicated intracellular cytokines for 15 min at room temperature.

### Quantitative Real-Time PCR

PCR primers and probes were designed with Universal ProbeLibrary Assay (Roche) or with TaqMan Gene Expression Assays (Applied Biosystems). Sequence of primers and probes in the latter case are shown in Table S1. PCR was performed with the TaqMan universal master mix with ROX (Applied Biosystems) according to the protocol provided. ABI PRISM7900HT Fast system (Applied Biosystems) or Biomark system (Fludigm) was used for quantitative real-time PCR according to the manufacturer's instructions. To ensure the specificity of the amplification products, a melting curve analysis was performed. Results were normalized and analyzed by ΔCt or ΔΔCt methods using the internal control gene *Hprt1*.

### Correlation Analysis of Microarrays

Gene expression detected using microarrays was normalized by the quantile normalization method [56]. Pearson's correlation values of logarithms of all signal intensities from 45,101 probes were calculated, and we performed hierarchal clustering of correlation matrices to indicate the degree of similarity between cell types. Scatter diagrams were drawn to display how similarly or differently genes were expressed in two samples. These diagrams contain only probes whose signals were present and coefficient values were shown in the figures.

### RSV-Induced AHR

Strain A2 of human RSV was used in this study. The general protocol for analyzing airway remodeling during RSV infection in mice is as follows: AHR was induced by sensitizing and challenging with OVA/alum (3–4 times) and/or infection with RSV (3–4 times), and then challenging with OVA, resulting in the examination of various pathological endpoints as previously described [57]–[59]. In the present study, we modified these protocols in order to analyze the physiological role of *i*NKT cells in the development of AHR mediated by RSV. In brief, mice were i.n. administered with RSV (10^6^ pfu) or PBS as a control 4 times at 10-d intervals. Mice were i.p. immunized with rec Gs/alum (50 µg/2 mg) 4 d after first RSV infections. Three days after the last RSV administration, mice were exposed i.n. to rec Gs recombinant protein and AHR responses were measured 1 d later.

### Measurement of Airway Responsiveness

Airway function was measured for changes in lung resistance (R_L_) and dynamic compliance in response to increasing doses of inhaled methacholine (1.25, 2.5, 5, 10, and 20 mg/ml) by using an invasive FlexiVent (SCIREQ Scientific Respiratory Equipment Inc.).

### Lymphocyte Isolation and Analysis of BAL Fluid

After measurement of AHR and sacrifice, the mouse trachea was cannulated, the lungs were lavaged twice with 1 ml PBS (10-fold PBS dilution), and the BAL fluid was pooled as previously described [30]. Lymphocytes from thymus, spleen, liver, lung, BM, inguinal LN, and mesenteric LN were isolated as described previously [53].

### Statistical Analysis

The statistical significance of differences was determined by *t* test, analysis of variance (ANOVA), or the Kruskal-Wallis test. The values were expressed as means ± SEM from independent experiments. Any differences with a *p* value of <0.05 were considered significant (* *p*<0.05; ** *p*<0.01).

## Supporting Information

Figure S1 Generation of *Il17rb* ^−/−^ mice. (A) Targeting strategy to disrupt the *il17rb* gene. Exons 1 and 2 were substituted with a neomycin resistance gene. Neo, neomycin; TK, thymidine kinase. (B) Genomic PCR analysis of offspring from the heterozygote intercrosses. Genomic DNA was extracted from mouse tails, amplified with primers indicated in (A). Genomic PCR results gave a single 500 bp band for wild-type (+/+), a 300 bp band for homozygous (−/−) and both bands for heterozygous mice (+/−).
(TIF)

Figure S2 Global gene expression profile in thymic *i*NKT subtypes. (A, B) Tree view representation of clustering analysis among the four *i*NKT subtypes in thymus from WT B6 (A) and between CD4^−^ or CD4^+^ IL-17RB^+^ cells from WT B6 or *Il15* ^L117P^ mice (B). The values represent coefficients between the indicated panels. *r* ^2^>0.9 in red and *r* ^2^<0.9 in orange.
(TIF)

Figure S3 In vitro cytokine production by thymic *i*NKT cells from *Il17rb* ^−/−^ and *Il15* ^L117P^ mice. Sorted *i*NKT cells (5×10^4^/100 µL) from thymus of WT B6, *Il17rb* ^−/−^, and *Il15* ^L117P^ mice were co-cultured with BM-DCs (5×10^3^/100 µL) for 48 h in the presence of indicated doses of α-GalCer. IFN-γ levels from *Il17rb* ^−/−^ *i*NKT cells were comparable to controls, whereas T_H_2 and T_H_17 cytokine levels were severely impaired. By contrast, IFN-γ from *Il15* ^L117P^ *i*NKT cells was markedly reduced, whereas T_H_2 and T_H_17 cytokine levels remained constant, which is the same outcome as in *i*NKT cells from spleen as shown in Figure 1.
(TIF)

Figure S4 Thymic *i*NKT cell subtypes in BALB/c mice. (A) FACS profile of MACS enriched α-GalCer/CD1d dimer^+^ TCRβ^+^ cells from B6 (upper) or BALB/c (lower) mice were further analyzed for the expression of the indicated markers. (B, C) Tree view representation of clustering analysis among the four thymic *i*NKT cell subtypes in BALB/c mice (B) and in comparison with thymic *i*NKT cell subtypes in B6 mice (C). The values represent coefficients between indicated panels. *r* ^2^>0.9 in red and 0.9<*r* ^2^ in orange.
(TIF)

Figure S5 Differential gene expression and cytokine production among thymic *i*NKT cell subtypes from BALB/c mice. (A, B, D, H) Quantitative RT-PCR analysis of thymic *i*NKT subtypes. Thymic *i*NKT cells were further divided into four subtypes based on the expression of IL-17RB and CD4. The results are shown as ΔCt. One representative out of three experiments is shown (mean ± SEM). (A) Purity of sorted cells was high based on the levels of *Il17rb* and *Cd4* mRNA expression. *Il2rb* ( = *Cd122*) expression was restricted to IL-17RB^−^ *i*NKT cells. (B) Expression of T_H_1/T_H_2/T_H_17 related genes. T_H_1: *Ifng*, *Tbx21* and *Stat4*, T_H_2:*Il4* and *Gata3*, and T_H_17: *Il17a*, *Il22* and *Rorc*. (D) Expression of cytokine receptor genes. Receptor for IL-12, IL-23, and IL-25 were analyzed. IL-12 receptor consists of IL-12Rβ2/IL-12Rβ1; IL-23 receptor: IL-23R/IL-12Rβ1; IL-25 receptor: IL-17RB/IL-17RA. (H) Expression of chemokine receptor genes. *Ccr4*, *Ccr6*, *Ccr7*, *Cxcr3*, and *Cxcr6* were analyzed. (C, E, F, G) Cytokine production by thymic *i*NKT cell subtypes in vitro. Sorted thymic *i*NKT subtypes (5×10^4^ cells/100 µL) were co-cultured with BM-DCs (5×10^3^/100 µL) for 48 h in the presence of α-GalCer (100 ng/µL) (C), IL-12 (10 ng/µL) (E), IL-23 (10 ng/µL) (F), or IL-25 (10 ng/µL) (G). Levels of IFN-γ, IL-4, IL-9, IL-10, IL-13, IL-17A, and IL-22 were analyzed. The data are representative of three independent experiments (mean ± SEM).
(TIF)

Figure S6 Cytokine gene expression in *i*NKT cells after being co-cultured with FT lobes. (A, B) Quantitative RT-PCR analysis of thymic *i*NKT precursors developed from Stage 1 (A) and Stage 2 (B) precursors. Cells shown in Figure 2H and 2I were sorted and analyzed the expression of indicated genes. The results are shown as ΔCt. One representative out of three experiments is shown (mean ± SEM).
(TIF)

Figure S7 Potential of cytokine production from *i*NKT cell subtypes. (A, B) The four *i*NKT subtypes (i.e. IL-17RB^+/−^ and CD4^+/−^) from thymus (A) and spleen (B) were sorted and treated with PMA and ionomycin. Indicated cytokines produced from each subtype were analyzed by intracellular cytokine staining. IFN-γ were highly produced from IL-17RB^−^ cells, while IL-10, IL-13 were from CD4^+^ IL-17RB^+^ subtypes, and IL-17A was from CD4^−^ IL-17RB^+^. All four subtypes had a potential to produce IL-4. The data are representative of three independent experiments.
(TIF)

Figure S8 Peripheral *i*NKT cell subtypes in BALB/c mice. (A, B) FACS profiles of peripheral *i*NKT cells in BALB/c mice. α-GalCer/CD1d dimer^+^ TCRβ^+^ *i*NKT cells (A), and *i*NKT subtypes based on the expression of IL-17RB and CD4 (B) in spleen, liver, bone marrow, lung, inguinal LN, and mesenteric LN in WT and *Il17rb* ^−/−^ mice. Numbers indicate percentage of total mononuclear cells (A) and *i*NKT cells (B).
(TIF)

Figure S9 Peripheral *i*NKT cell subtypes in *Il15* ^L117P^ mice. (A, B) Number of total *i*NKT cells (A) and *i*NKT subtypes based on the expression of IL-17RB and CD4 (B) in WT B6 and *Il15* ^L117P^ mice. Both CD4^−^ and CD4^+^, IL-17RB^−^ *i*NKT cells in *Il15* ^L117P^ mice were dramatically reduced in number among all of the tested organs. The majority of *i*NKT cells in liver and BM were IL-17RB^−^ *i*NKT cells, resulting in decreased cell numbers in *Il15* ^L117P^ mice. In contrast, both CD4^−^ and CD4^+^ IL-17RB^+^ *i*NKT cells in *Il15* ^L117P^ mice were present in numbers comparable to WT B6.
(TIF)

Figure S10 Quantitative RT-PCR analysis of *i*NKT subtypes in the periphery. (A–D) *i*NKT cell subtypes from the tissues shown in Figure 4B were sorted and the same genes were analyzed as in Figures 3A, 3B, 3D, and 3H. (A) The purity of sorted cells was high based on the respective level of *Cd4* and *Il17rb* mRNA expression. *Il2rb* ( = *Cd122*) expression was restricted to IL-17RB^−^ *i*NKT cells. (B–D) Expression of T_H_1/T_H_2/T_H_17 related genes (B), cytokine receptor genes (C), and chemokine receptor genes (D).
(TIF)

Figure S11 Cytokine production by splenic *i*NKT cell subtypes from BALB/c mice in vitro. (A–D) Sorted splenic *i*NKT subtypes (5×10^4^ cells/100 µL) were co-cultured with BM-DCs (5×10^3^/100 µL) for 48 h in the presence of α-GalCer (100 ng/mL) (A), IL-12 (10 ng/mL) (B), IL-23 (10 ng/mL) (C), and IL-25 (10 ng/mL) (D). Levels of IFN-γ, IL-4, IL-9, IL-10, IL-13, IL-17A, and IL-22 were analyzed.
(TIF)

Figure S12 Expression of recombinant RSV-Gs protein. (A) Schematic representation of RSV-G proteins. Membrane form (Gm, upper) and soluble form (Gs, middle) of RSV-G were shown. Recombinant RSV-Gs protein (rec Gs) was expressed as a fusion with a mouse IL-2 leader sequence and a C-terminal tag (BirA-6His). (B) Western blot analysis of the culture supernatant after transfection of the rec Gs expression vector into HEK293 cells. Expressed rec Gs was biotinylated by the BirA enzyme and detected by stereptavidin-HRP. Rec Gs is a highly glycosylated mucin-like protein, resulting in a diffuse band in the region of 25–120 kDa.
(TIF)

Table S1 Primers and probes for quantitative real-time PCR used in this study.
(DOC)

## Abbreviations

α-GalCer: α-Galactosylceramide
AHR: airway hyperreactivity
BAL: bronchoalveolar lavage
*i*NKT: invariant natural killer T
ROR: retinoic acid receptor-related orphan receptor
RSV: respiratory syncytial virus
