# Supplementary figures and images for: Development and Function of Invariant Natural Killer T Cells Producing TH2- and TH17-Cytokines
Source: PLoS Biol. 2012 Feb 7;10(2):e1001255. doi: 10.1371/journal.pbio.1001255 (PMC3274505; doi:10.1371/journal.pbio.1001255)

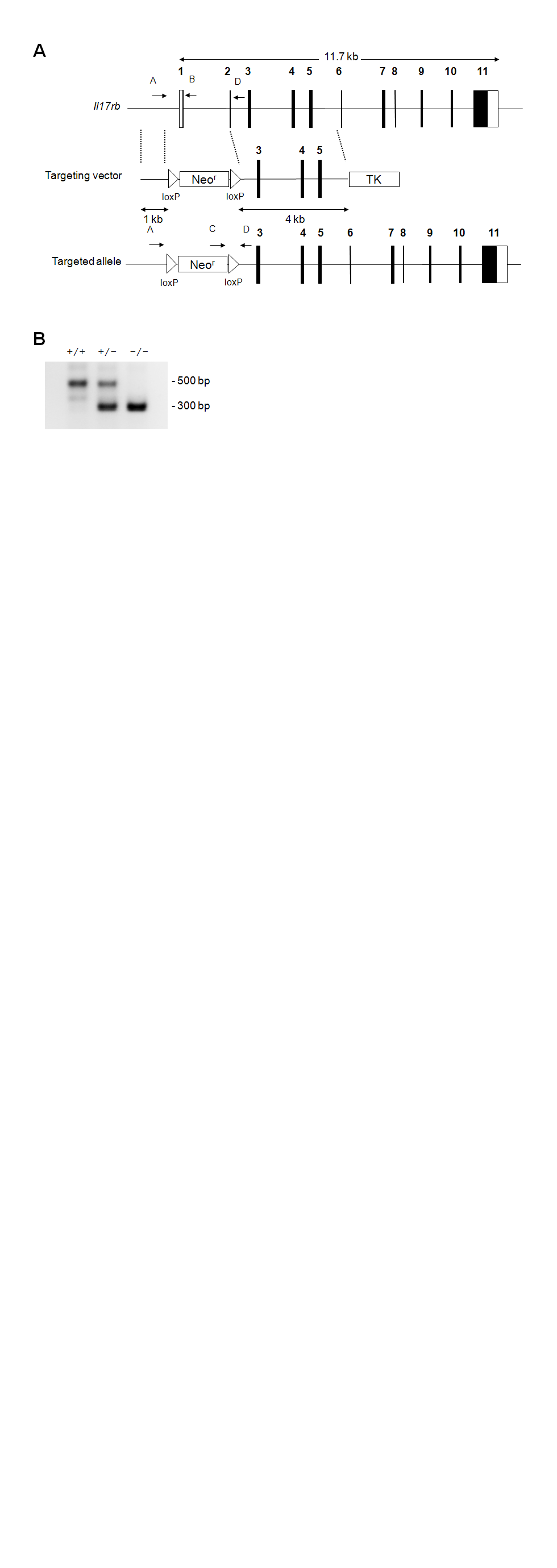

Supplement: Figure S1 — Generation of Il17rb −/− mice. (A) Targeting strategy to disrupt the il17rb gene. Exons 1 and 2 were substituted with a neomycin resistance gene. Neo, neomycin; TK, thymidine kinase. (B) Genomic PCR analysis of offspring from the heterozygote intercrosses. Genomic DNA was extracted from mouse tails, amplified with primers indicated in (A). Genomic PCR results gave a single 500 bp band for wild-type (+/+), a 300 bp band for homozygous (−/−) and both bands for heterozygous mice (+/−). (TIF) [file pbio.1001255.s001.tif]

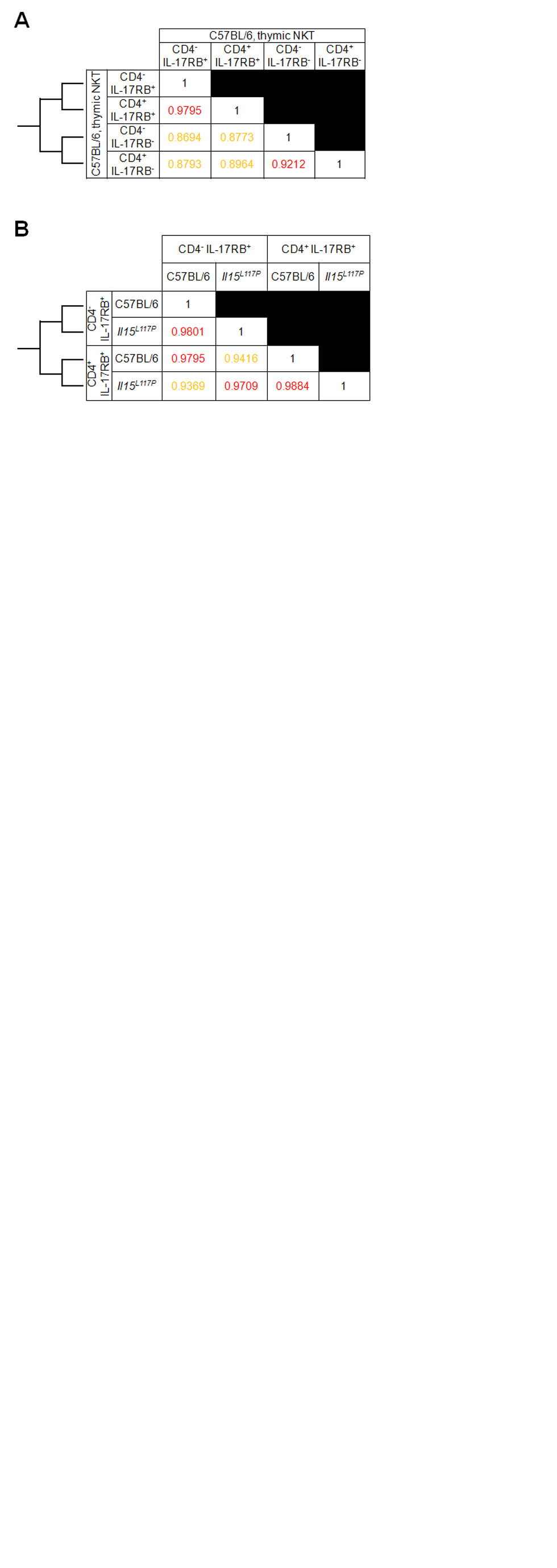

Supplement: Figure S2 — Global gene expression profile in thymic iNKT subtypes. (A, B) Tree view representation of clustering analysis among the four iNKT subtypes in thymus from WT B6 (A) and between CD4− or CD4+ IL-17RB+ cells from WT B6 or Il15 L117P mice (B). The values represent coefficients between the indicated panels. r 2>0.9 in red and r 2<0.9 in orange. (TIF) [file pbio.1001255.s002.tif]

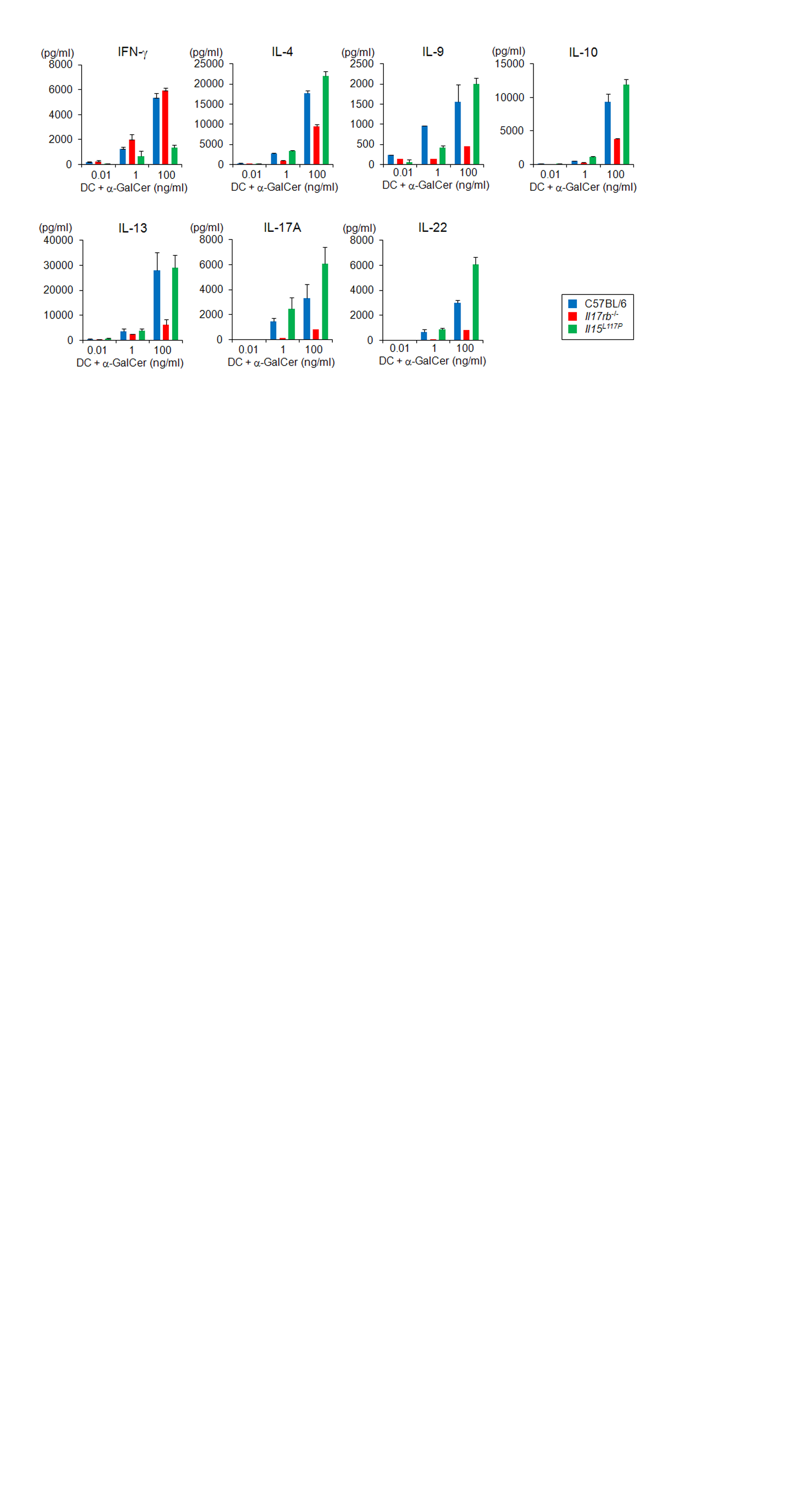

Supplement: Figure S3 — In vitro cytokine production by thymic iNKT cells from Il17rb −/− and Il15 L117P mice. Sorted iNKT cells (5×104/100 µL) from thymus of WT B6, Il17rb −/−, and Il15 L117P mice were co-cultured with BM-DCs (5×103/100 µL) for 48 h in the presence of indicated doses of α-GalCer. IFN-γ levels from Il17rb −/− iNKT cells were comparable to controls, whereas TH2 and TH17 cytokine levels were severely impaired. By contrast, IFN-γ from Il15 L117P iNKT cells was markedly reduced, whereas TH2 and TH17 cytokine levels remained constant, which is the same outcome as in iNKT cells from spleen as shown in Figure 1. (TIF) [file pbio.1001255.s003.tif]

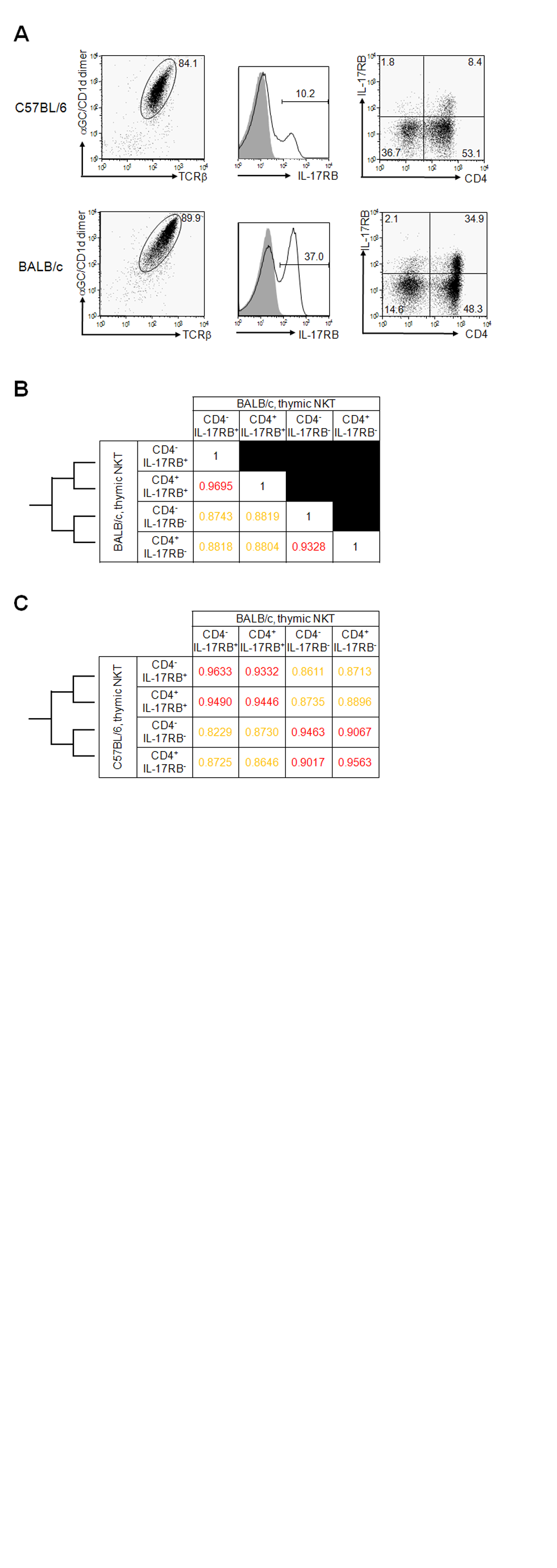

Supplement: Figure S4 — Thymic iNKT cell subtypes in BALB/c mice. (A) FACS profile of MACS enriched α-GalCer/CD1d dimer+ TCRβ+ cells from B6 (upper) or BALB/c (lower) mice were further analyzed for the expression of the indicated markers. (B, C) Tree view representation of clustering analysis among the four thymic iNKT cell subtypes in BALB/c mice (B) and in comparison with thymic iNKT cell subtypes in B6 mice (C). The values represent coefficients between indicated panels. r 2>0.9 in red and 0.9<r 2 in orange. (TIF) [file pbio.1001255.s004.tif]

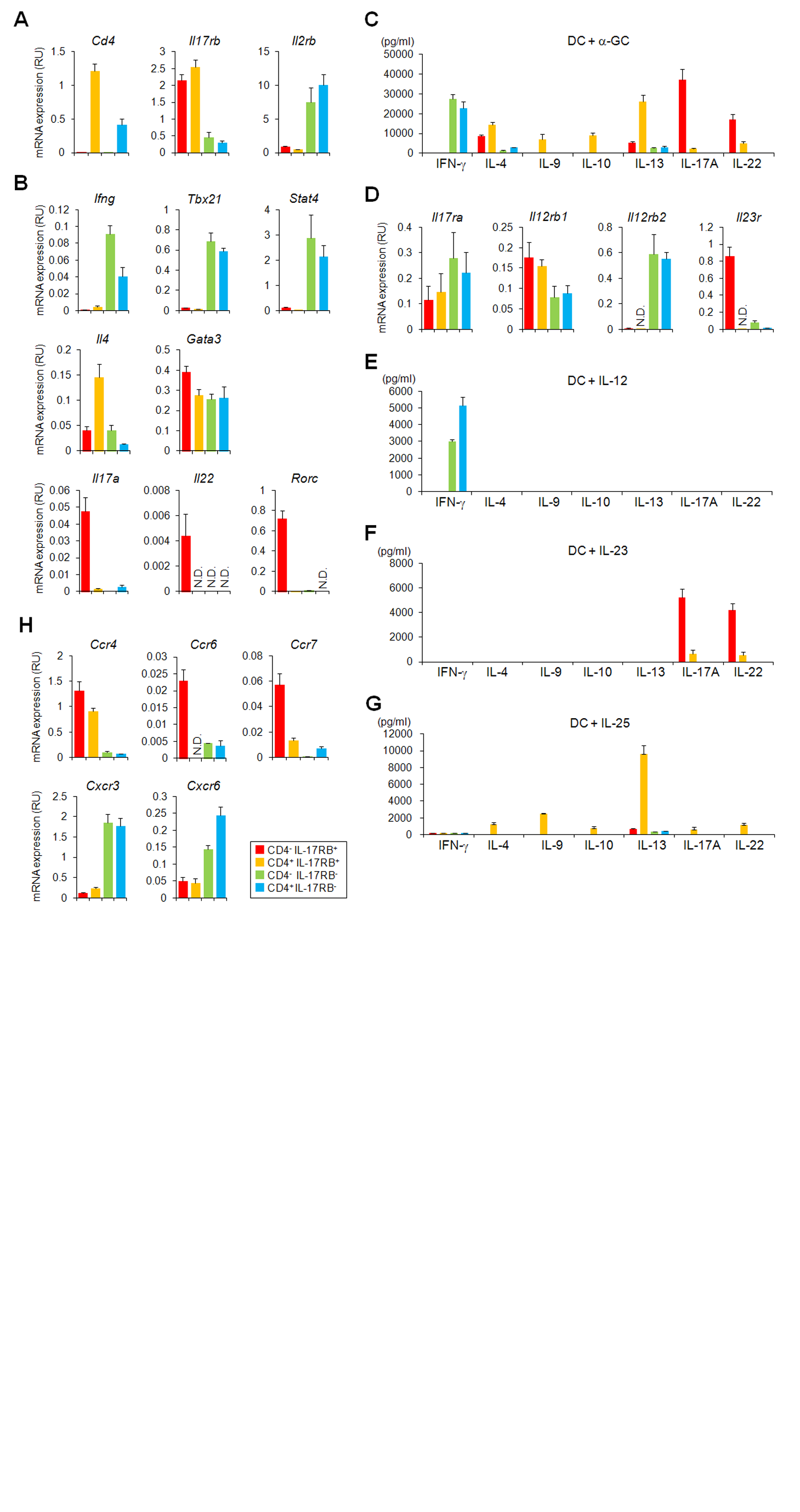

Supplement: Figure S5 — Differential gene expression and cytokine production among thymic iNKT cell subtypes from BALB/c mice. (A, B, D, H) Quantitative RT-PCR analysis of thymic iNKT subtypes. Thymic iNKT cells were further divided into four subtypes based on the expression of IL-17RB and CD4. The results are shown as ΔCt. One representative out of three experiments is shown (mean ± SEM). (A) Purity of sorted cells was high based on the levels of Il17rb and Cd4 mRNA expression. Il2rb ( = Cd122) expression was restricted to IL-17RB− iNKT cells. (B) Expression of TH1/TH2/TH17 related genes. TH1: Ifng, Tbx21 and Stat4, TH2:Il4 and Gata3, and TH17: Il17a, Il22 and Rorc. (D) Expression of cytokine receptor genes. Receptor for IL-12, IL-23, and IL-25 were analyzed. IL-12 receptor consists of IL-12Rβ2/IL-12Rβ1; IL-23 receptor: IL-23R/IL-12Rβ1; IL-25 receptor: IL-17RB/IL-17RA. (H) Expression of chemokine receptor genes. Ccr4, Ccr6, Ccr7, Cxcr3, and Cxcr6 were analyzed. (C, E, F, G) Cytokine production by thymic iNKT cell subtypes in vitro. Sorted thymic iNKT subtypes (5×10^4^ cells/100 µL) were co-cultured with BM-DCs (5×103/100 µL) for 48 h in the presence of α-GalCer (100 ng/µL) (C), IL-12 (10 ng/µL) (E), IL-23 (10 ng/µL) (F), or IL-25 (10 ng/µL) (G). Levels of IFN-γ, IL-4, IL-9, IL-10, IL-13, IL-17A, and IL-22 were analyzed. The data are representative of three independent experiments (mean ± SEM). (TIF) [file pbio.1001255.s005.tif]

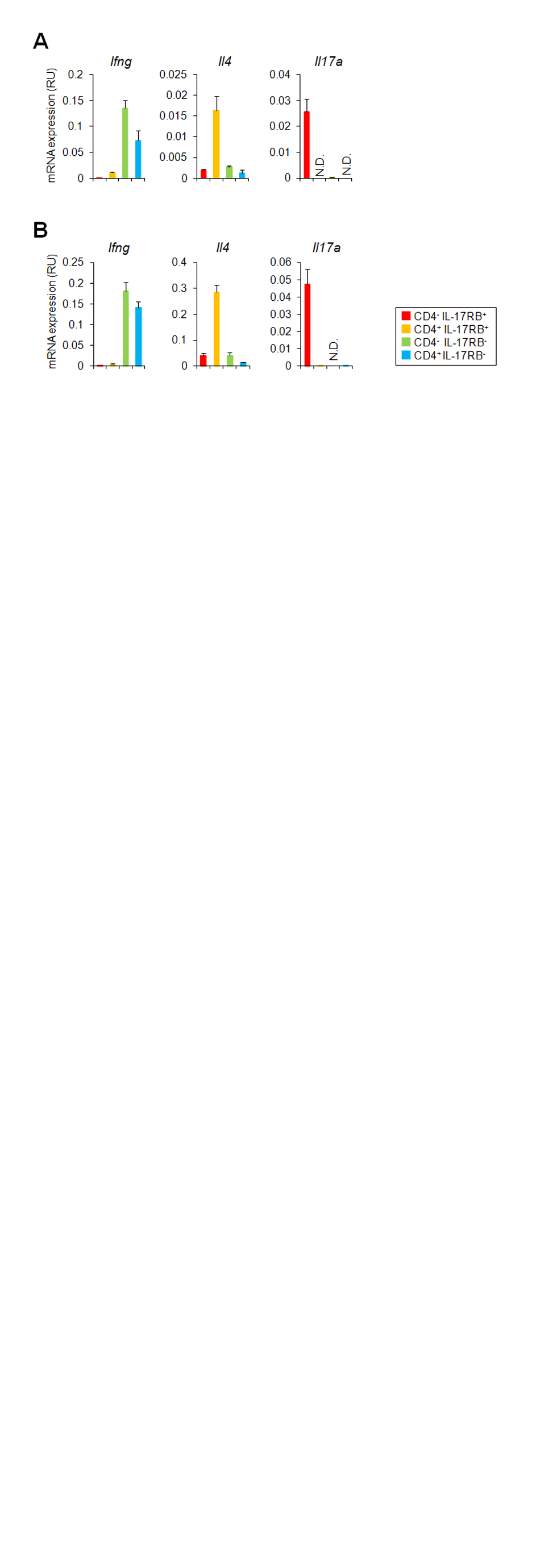

Supplement: Figure S6 — Cytokine gene expression in iNKT cells after being co-cultured with FT lobes. (A, B) Quantitative RT-PCR analysis of thymic iNKT precursors developed from Stage 1 (A) and Stage 2 (B) precursors. Cells shown in Figure 2H and 2I were sorted and analyzed the expression of indicated genes. The results are shown as ΔCt. One representative out of three experiments is shown (mean ± SEM). (TIF) [file pbio.1001255.s006.tif]

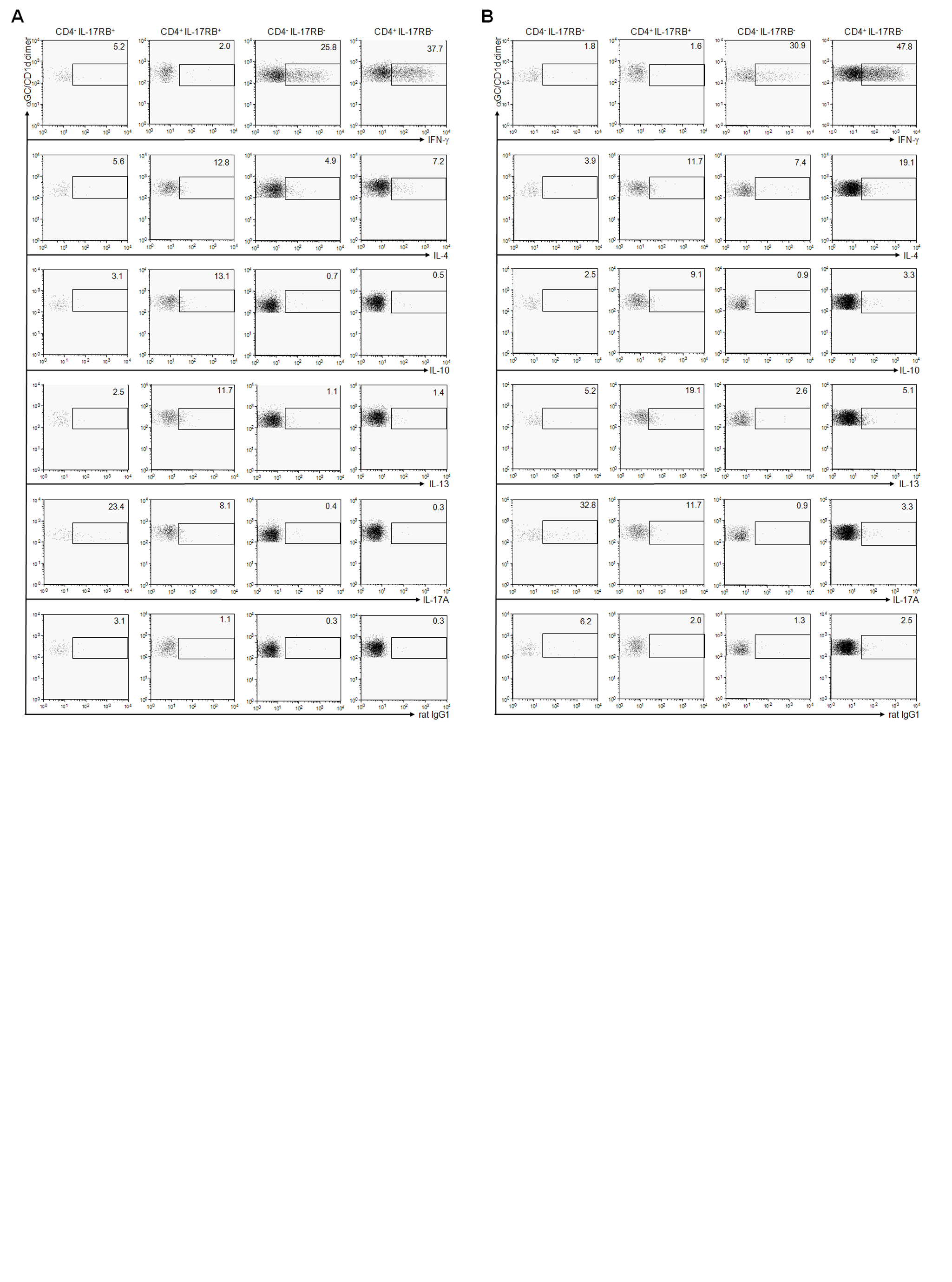

Supplement: Figure S7 — Potential of cytokine production from iNKT cell subtypes. (A, B) The four iNKT subtypes (i.e. IL-17RB+/− and CD4+/−) from thymus (A) and spleen (B) were sorted and treated with PMA and ionomycin. Indicated cytokines produced from each subtype were analyzed by intracellular cytokine staining. IFN-γ were highly produced from IL-17RB− cells, while IL-10, IL-13 were from CD4+ IL-17RB+ subtypes, and IL-17A was from CD4− IL-17RB+. All four subtypes had a potential to produce IL-4. The data are representative of three independent experiments. (TIF) [file pbio.1001255.s007.tif]

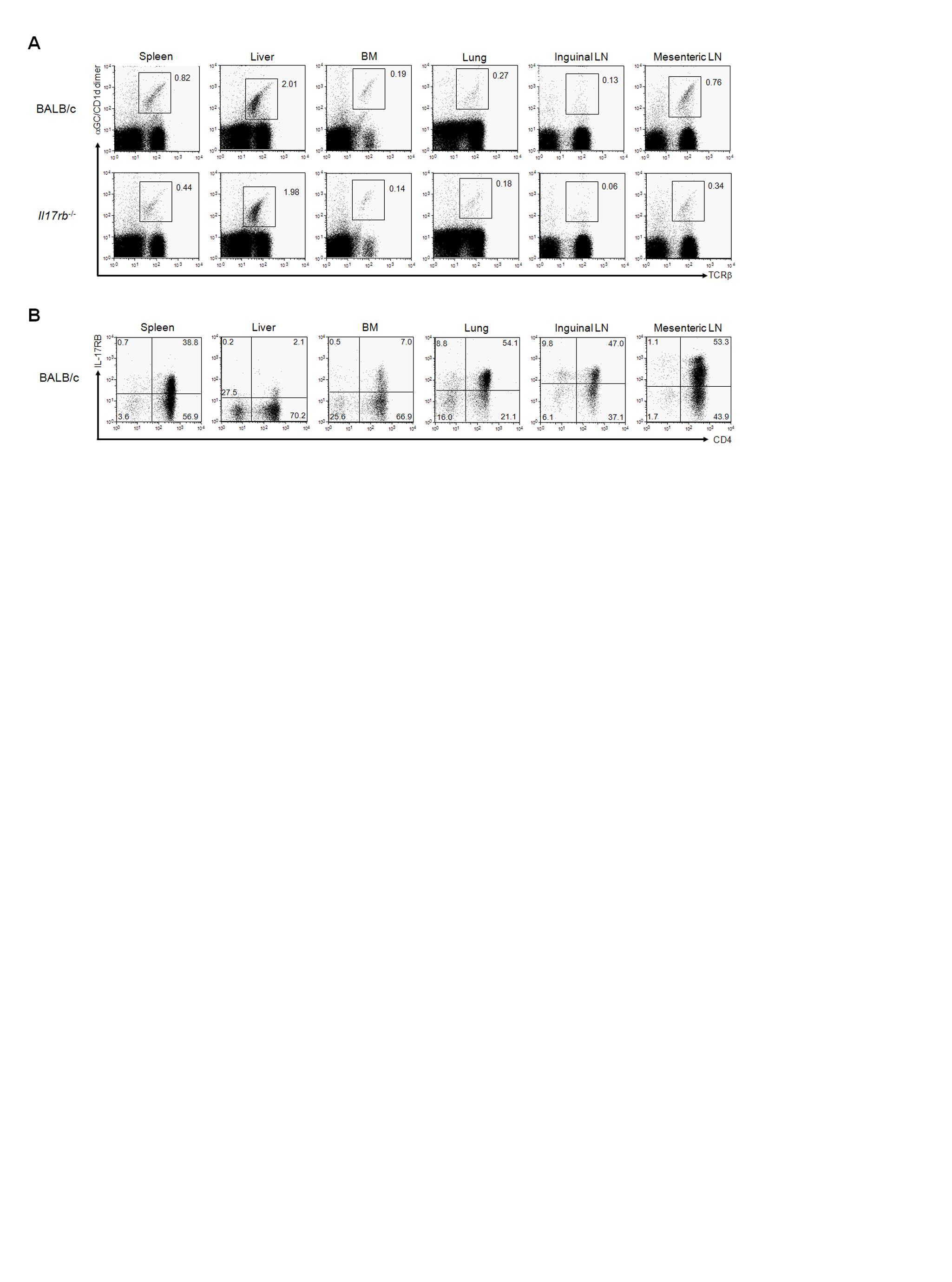

Supplement: Figure S8 — Peripheral iNKT cell subtypes in BALB/c mice. (A, B) FACS profiles of peripheral iNKT cells in BALB/c mice. α-GalCer/CD1d dimer+ TCRβ+ iNKT cells (A), and iNKT subtypes based on the expression of IL-17RB and CD4 (B) in spleen, liver, bone marrow, lung, inguinal LN, and mesenteric LN in WT and Il17rb −/− mice. Numbers indicate percentage of total mononuclear cells (A) and iNKT cells (B). (TIF) [file pbio.1001255.s008.tif]

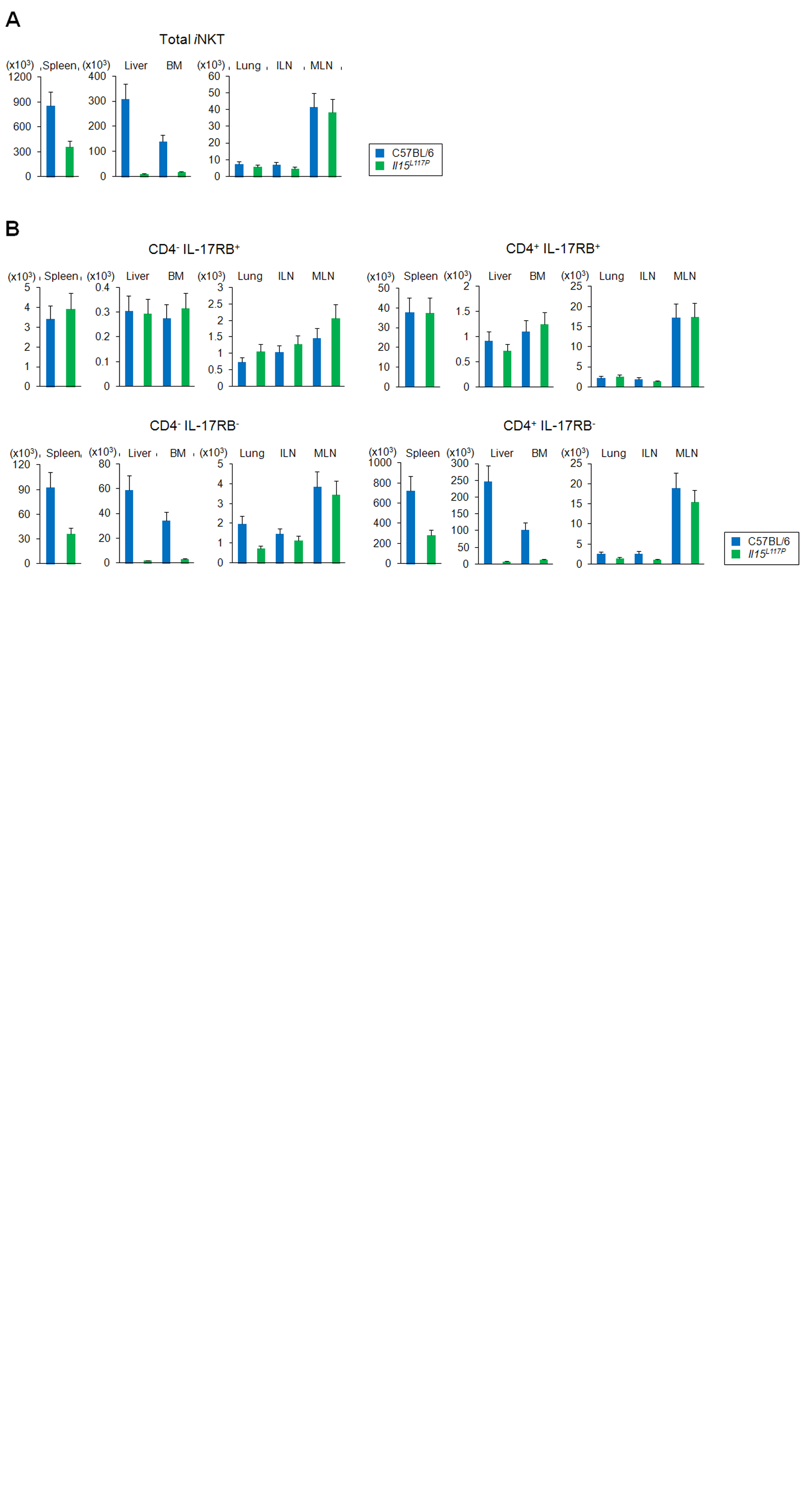

Supplement: Figure S9 — Peripheral iNKT cell subtypes in Il15 L117P mice. (A, B) Number of total iNKT cells (A) and iNKT subtypes based on the expression of IL-17RB and CD4 (B) in WT B6 and Il15 L117P mice. Both CD4− and CD4+, IL-17RB− iNKT cells in Il15 L117P mice were dramatically reduced in number among all of the tested organs. The majority of iNKT cells in liver and BM were IL-17RB− iNKT cells, resulting in decreased cell numbers in Il15 L117P mice. In contrast, both CD4− and CD4+ IL-17RB+ iNKT cells in Il15 L117P mice were present in numbers comparable to WT B6. (TIF) [file pbio.1001255.s009.tif]

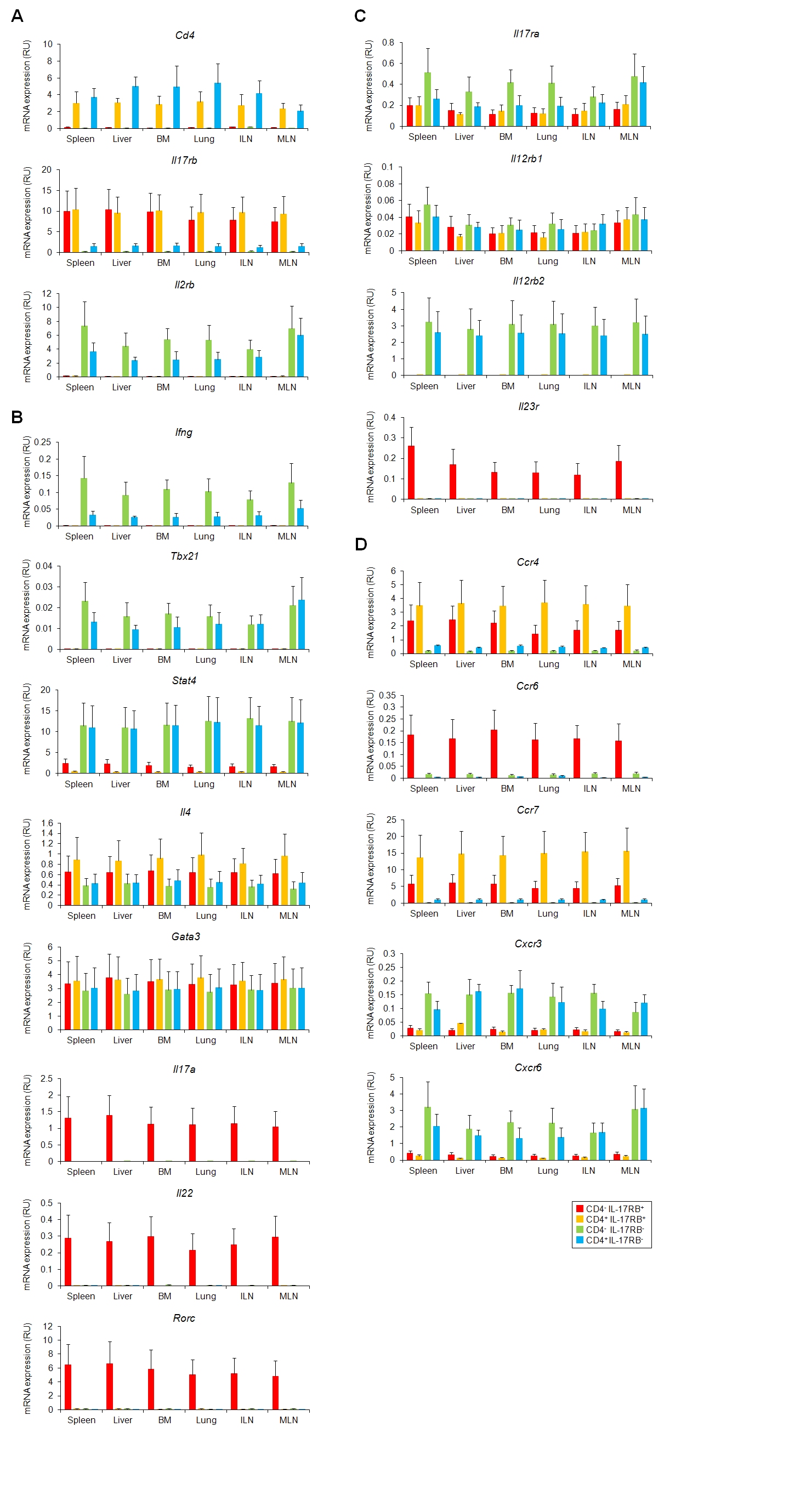

Supplement: Figure S10 — Quantitative RT-PCR analysis of iNKT subtypes in the periphery. (A–D) iNKT cell subtypes from the tissues shown in Figure 4B were sorted and the same genes were analyzed as in Figures 3A, 3B, 3D, and 3H. (A) The purity of sorted cells was high based on the respective level of Cd4 and Il17rb mRNA expression. Il2rb ( = Cd122) expression was restricted to IL-17RB− iNKT cells. (B–D) Expression of TH1/TH2/TH17 related genes (B), cytokine receptor genes (C), and chemokine receptor genes (D). (TIF) [file pbio.1001255.s010.tif]

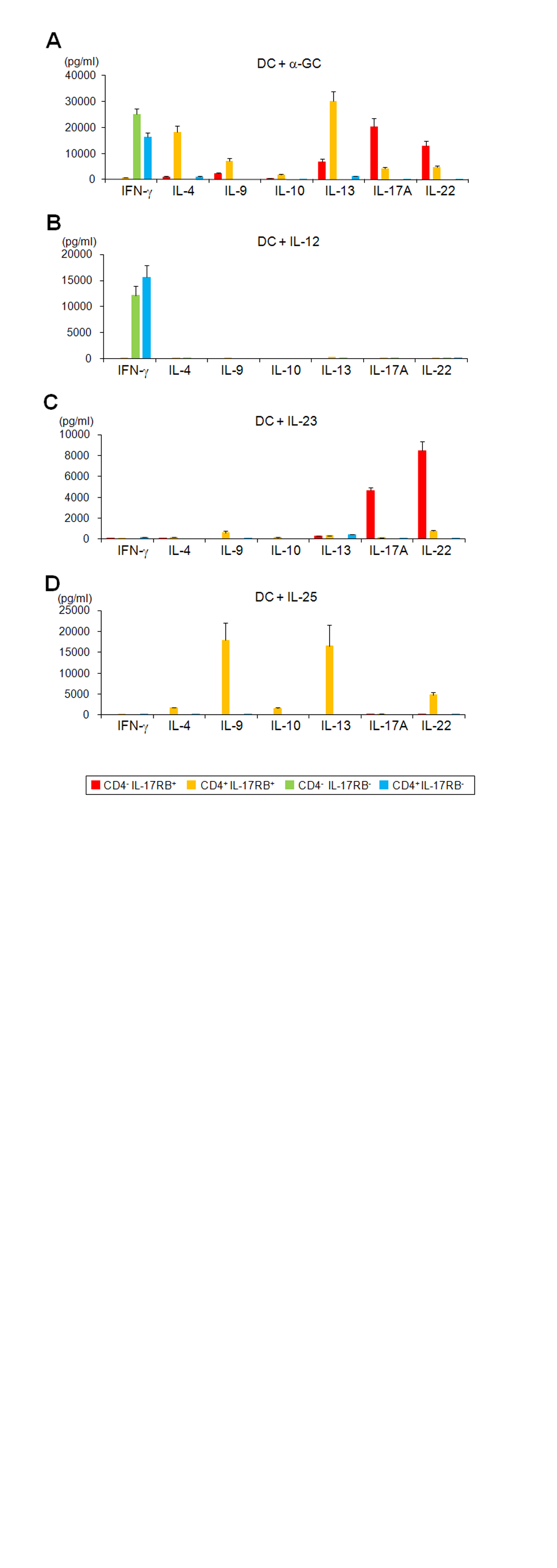

Supplement: Figure S11 — Cytokine production by splenic iNKT cell subtypes from BALB/c mice in vitro. (A–D) Sorted splenic iNKT subtypes (5×10^4^ cells/100 µL) were co-cultured with BM-DCs (5×103/100 µL) for 48 h in the presence of α-GalCer (100 ng/mL) (A), IL-12 (10 ng/mL) (B), IL-23 (10 ng/mL) (C), and IL-25 (10 ng/mL) (D). Levels of IFN-γ, IL-4, IL-9, IL-10, IL-13, IL-17A, and IL-22 were analyzed. (TIF) [file pbio.1001255.s011.tif]

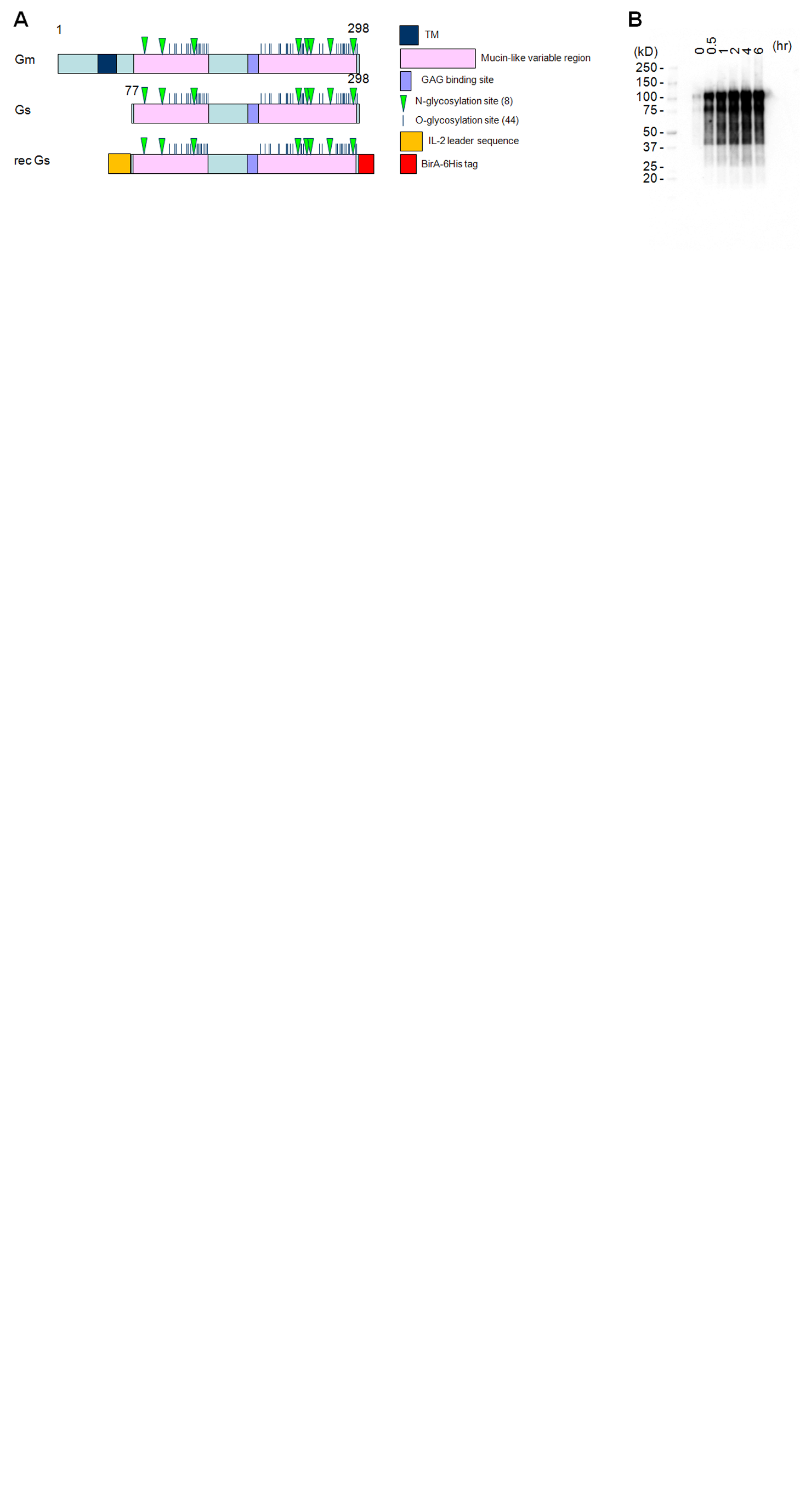

Supplement: Figure S12 — Expression of recombinant RSV-Gs protein. (A) Schematic representation of RSV-G proteins. Membrane form (Gm, upper) and soluble form (Gs, middle) of RSV-G were shown. Recombinant RSV-Gs protein (rec Gs) was expressed as a fusion with a mouse IL-2 leader sequence and a C-terminal tag (BirA-6His). (B) Western blot analysis of the culture supernatant after transfection of the rec Gs expression vector into HEK293 cells. Expressed rec Gs was biotinylated by the BirA enzyme and detected by stereptavidin-HRP. Rec Gs is a highly glycosylated mucin-like protein, resulting in a diffuse band in the region of 25–120 kDa. (TIF) [file pbio.1001255.s012.tif]
