# Supplementary material for: Development and Function of Invariant Natural Killer T Cells Producing TH2- and TH17-Cytokines
Source: PLoS Biol. 2012 Feb 7;10(2):e1001255. doi: 10.1371/journal.pbio.1001255 (PMC3274505; doi:10.1371/journal.pbio.1001255)
Supplement: Table S1 — Primers and probes for quantitative real-time PCR used in this study. (DOC) [file pbio.1001255.s013.doc]

**Supplementary Table**

**Table S1. Primers and probes for quantitative real time PCR used in this study.**

| Gene |  | Sequence |
| --- | --- | --- |
| *Cd4* | Forward primer | CTGACTCTGACTCTGGACAAAGG |
|  | Reverse primer | GGAGAGGTAGGTCCCATCACC |
|  | Probe | TTGAGCTGAGCCACTTTCATCACCACCA |
| *Il17rb* | Forward primer | CCAGATGACAACAGACGCATG |
|  | Reverse primer | GAGCATGGTGGAAATAGGAAAGG |
|  | Probe | CGTCTTCGTGCTCCTTCCTTGCCTCC |
| *Il2rb* | Forward primer | GAAGGGTTGGCGTAGGGTAAAG |
|  | Reverse primer | GCAGAACTTGGAGGGAATGAGG |
|  | Probe | TCCCTTTGACAACCTTCGCCTGGTGG |
| *Ifng* | Forward primer | GGATGCATTCATGAGTATTGCCAAG |
|  | Reverse primer | CTCCTTTTCCGCTTCCTGAGG |
|  | Probe | AGGTCAACAACCCACAGGTCCAGCG |
| *Tbx21* | Forward primer | AAGGATTCCGGGAGAACTTTGAG |
|  | Reverse primer | TGGTTGGATAGAAGAGGTGAGAAG |
|  | Probe | TGTACGCATCTGTTGATACGAGTGTCCCCT |
| *Stat4* | Forward primer | AGGGAAGAGAGGAGAATATTGGC |
|  | Reverse primer | GTTCCACATTCCTTTGTCTTTCAG |
|  | Probe | CAGCCAACATGCCTATCCAGGGACCT |
| *Il4* | Forward primer | CATCGGCATTTTGAACGAGGTC |
|  | Reverse primer | CGTTGCTGTGAGGACGTTTG |
|  | Probe | TCTCCGTGCATGGCGTCCCTTCTCC |
| *Gata3* | Forward primer | GCTACGGTGCAGAGGTATCC |
|  | Reverse primer | TCCAGCCAGGGCAGAGATC |
|  | Probe | CGACCCACCACGGGAGCCAGGT |
| *Il17a* | Forward primer | CCTTGGCGCAAAAGTGAGC |
|  | Reverse primer | ATATCTATCAGGGTCTTCATTGCG |
|  | Probe | ACTACCTCAACCGTTCCACGTCACCC |
| *Il22* | Forward primer | AGCTTGAGGTGTCCAACTTCC |
|  | Reverse primer | AACAGTTTCTCCCCGATGAGC |
|  | Probe | AGCCGTACATCGTCAACCGCACCT |
| *Rorc* | Forward primer | GGCTTTCCATCATCATCTCTGC |
|  | Reverse primer | GGTGGAGGTGCTGGAAGATC |
|  | Probe | CCTCCTAGCCAAGCTGCCACCCAAAG |
| *Il17ra* | Forward primer | GTGCCCTGCCCAGTAATCTC |
|  | Reverse primer | ATGGCGATGAGTGTGATGAGG |
|  | Probe | ACCACAGTTCCCAAGCCAGTTGCAGA |
| *Il12rb1* | Forward primer | CGCTGCGAGGCTGAAGAC |
|  | Reverse primer | CGCAGTCCGTCAAGTGTCAC |
|  | Probe | CACGAGCCACTCTGACTCCCACGC |
| *Il12rb2* | Forward primer | CGCTTCTGCACCCACTCAC |
|  | Reverse primer | TGCCAGGTCACTAGAATGTTGTC |
|  | Probe | CACTGGGTTGCTGGCTCCTCACCA |
| *Il23r* | Forward primer | GCTTCTACTACATTTGGGACATGAG |
|  | Reverse primer | CACCAGGCTCAACCCACATG |
|  | Probe | TGATTCCTCCGTGACACCATCTGAAGAGCA |
| *Ccr4* | Forward primer | CTCAGGATCACTTTCAGAAGAGC |
|  | Reverse primer | GGTGGTGTCTGTGACCTCTG |
|  | Probe | AGGCAGCTCAACTGTTCTCATTGGCT |
| *Ccr6* | Forward primer | CTGCCCACTTCCCTTTCTACAC |
|  | Reverse primer | CTGTGTTGTCATAATCATCCGTTCC |
|  | Probe | TCATTCCCCAGGCAGGCGTGGTTCT |
| *Ccr7* | Forward primer | CATGGACCCAGGGAAACCC |
|  | Reverse primer | TGACCTCATCTTGGCAGAAGC |
|  | Probe | TGACAAGGAGAGCCACCACCAGCACG |
| *Cxcr3* | Forward primer | GAAGCAGGCAGCACGAGAC |
|  | Reverse primer | CCGAGGCATCTAGCACTTGAC |
|  | Probe | CGGAGCACCAGCCAAGCCATGTACC |
| *Cxcr6* | Forward primer | CACACTTCACTCTGGAACAAAGC |
|  | Reverse primer | TGGCTGTTATCACTGGAATTGTTG |
|  | Probe | AGCCAGAAATCTCCCTCGTAGTGCCCATC |
| *E4bp4* | Forward primer | ACAGCCGCCCTTTCTTTTCC |
|  | Reverse primer | GGACTTCAGCCTCTCATCCATC |
|  | Probe | ACCAGGGAGCAGAACCACGATAACCC |
| *Hprt1* | Forward primer | GAGGATTTGGAAAAAGTGTTTATTCCTC |
|  | Reverse primer | GATGGCCTCCCATCTCCTTC |
|  | Probe | CATCTCGAGCAAGTCTTTCAGTCCTGTCCA |
